# Small molecule metabolites: discovery of biomarkers and therapeutic targets

**DOI:** 10.1038/s41392-023-01399-3

**Published:** 2023-03-20

**Authors:** Shi Qiu, Ying Cai, Hong Yao, Chunsheng Lin, Yiqiang Xie, Songqi Tang, Aihua Zhang

**Affiliations:** 1 International Advanced Functional Omics Platform, Scientific Experiment Center, Hainan General Hospital (Hainan Affiliated Hospital of Hainan Medical University), College of Chinese Medicine, Hainan Medical University, Xueyuan Road 3, Haikou, 571199 China; 2 Graduate School, Heilongjiang University of Chinese Medicine, Harbin, 150040 China; 3 First Affiliated Hospital, Harbin Medical University, Harbin, 150081 China; 4 Second Affiliated Hospital, Heilongjiang University of Chinese Medicine, Harbin, 150001 China

**Keywords:** Structural biology, Drug screening, Target identification

## Abstract

Metabolic abnormalities lead to the dysfunction of metabolic pathways and metabolite accumulation or deficiency which is well-recognized hallmarks of diseases. Metabolite signatures that have close proximity to subject’s phenotypic informative dimension, are useful for predicting diagnosis and prognosis of diseases as well as monitoring treatments. The lack of early biomarkers could lead to poor diagnosis and serious outcomes. Therefore, noninvasive diagnosis and monitoring methods with high specificity and selectivity are desperately needed. Small molecule metabolites-based metabolomics has become a specialized tool for metabolic biomarker and pathway analysis, for revealing possible mechanisms of human various diseases and deciphering therapeutic potentials. It could help identify functional biomarkers related to phenotypic variation and delineate biochemical pathways changes as early indicators of pathological dysfunction and damage prior to disease development. Recently, scientists have established a large number of metabolic profiles to reveal the underlying mechanisms and metabolic networks for therapeutic target exploration in biomedicine. This review summarized the metabolic analysis on the potential value of small-molecule candidate metabolites as biomarkers with clinical events, which may lead to better diagnosis, prognosis, drug screening and treatment. We also discuss challenges that need to be addressed to fuel the next wave of breakthroughs.

## Introduction

Metabolite biosignatures from human biofluids providing a link between genotype, environment and phenotype, are attractive biomarkers for the clinical diagnosis, prognosis, and diseases classification.^1–8^ It can provide a unique metabolic readout and snapshot of the health/disease status of key information about the downstream products related to various metabolic processes.^9–12^ Differential metabolites can improve the specificity and accuracy as biomarkers for patient diagnosis, patient monitoring, risk prediction and prognosis.^13–16^ Discovery and identification of small molecule metabolites or metabolic pathway alterations is useful for understanding the pathophysiology of diseases, and help identify therapeutic targets.^17–27^ Metabolome represent the upstream input from environment and downstream output of genome, the collection of bioactive small molecule metabolites including nucleotides, carbohydrates, amino acid, and fatty acid, has used for discovery of early prediction and diagnosis biomarkers of diseases that insight into the best use of interventions.^28–35^ Endogenous metabolites could provide unique metabolic insights into the mechanistic basis and therapeutic targets of disease and also leads to personalized metabolic phenotype.^36^

**Fig. 1:**
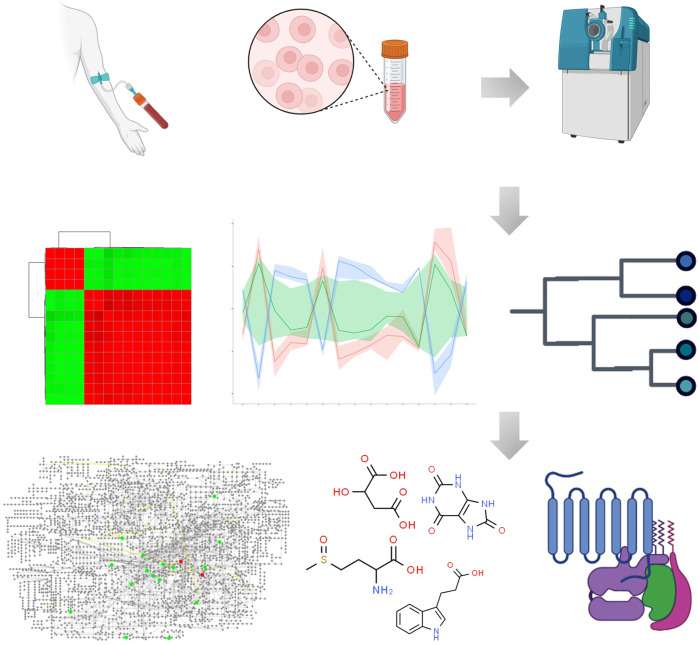
Analytical workflow of small molecule metabolites-based metabolomics. The first stage involves experimental design, followed by election of biological subjects, sample collection, preparation, and metabolite extraction. Next is acquisition and processing of data, then data analysis, and finally, making sense of the data through biomarker discovery, and functional interpretation. The images were obtained using the example data provided by the MetaboAnalyst 5.0 and figures created by BioRender

Bioactive functions and detail molecular mechanisms of small metabolites have gradually raised attention of scientists and researchers.^37–43^ Fortunately, advancements in metabolomics technologies hold promise as non-invasive and high-throughput tool that conventionally divided into untargeted and untargeted analysis has demonstrated high value in investigation of metabolite signatures, and allowed researcher to establish mass spectrometry-based comprehensive profiling of small molecule metabolites to provide insight into metabolic function.^44–55^. Metabolomics, the science of characterizing known and unknown small molecule metabolites, appears to be an ideally tool for disease characterization and monitoring as well as the investigation of disease pathophysiology and biochemical characteristics in body systems.^56–60^ Major approaches include metabolic phenotyping, metabolic fingerprinting, metabolic profiling and targeted metabolite analysis.^61–75^ Fig. 1 shows general workflow for biomarker discovery from small-molecule metabolites through metabolomics approach. Metabolic phenotypes could reflect the metabolic response feature variation to pathophysiological stimuli at a certain time point.^76–78^ According to specific profiles, metabolic regulation associated with therapeutic responses is new therapeutic strategy for diseases.^79–81^ Since metabolomics aims to identify small metabolites from biological system, insights into metabolism and its regulation mechanisms that symptom generation and therapeutic response, provides an innovative approach to answer phenotype-related questions distinctly altered in diseases, elucidate the biochemical functions and delineate the associated mechanisms implicated in the dysregulated metabolism from patients within clinical settings.^82–92^

Identification of the small metabolites and molecular mechanisms using high-throughput metabolomics may allow for the rapid development of biomarker and improving disease diagnosis, prognosis, treatment response, and for revealing mechanisms and disease etiology, therapeutic target for ameliorating the quality of life in patients.^93–99^ Using small molecule metabolites-based metabolomics for discovery of metabolic biomarkers to diagnosis and then providing key information for biomarker validation and elucidation of the molecular mechanism of disease, has attracted broad interest.^100,101^ This review focused on functional features of small molecule metabolites, utility of them as biomarkers and therapeutic targets for disease via the function relationship and associated molecular mechanisms, and also discussed its progress in the early diagnosis, prognosis, and pathogenesis of disease at the level of metabolism in vivo, which is expected to translate the milestone findings into clinical trials to enhance the efficacy and provide new sights for human clinical use in the future.

## Advanced technology platform

**Fig. 2:**
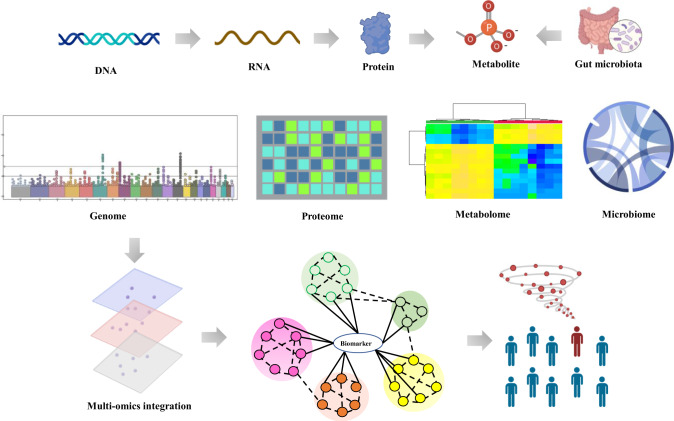
Schematic representation of the most commonly used omic platforms for multi-omics studies. Metabolites are the downstream products of the genome, transcriptome, proteome, and enzymatic reactions, which are also affected by environmental exposures. The metabolome provides a functional readout of these upstream changes. Multi-omics (including genome, transcriptome, proteome, metabolome, and microbiome data) are collected from patients and integrated to identify personalized functional signatures using complex and comprehensive network analysis. The figures created by BioRender

Metabolites are the final downstream products of protein translation and gene transcription or cellular perturbations to the proteome, genome or transcriptome, have potentially crucial linkage between genotype and environment, and provide a closer image of the final phenotype.^102–105^ A human metabolome mainly contains the detailed information of 41,993 small-molecule metabolites, has been implemented for public.^106,107^ Metabolites act as signaling molecules, serve as cofactors, energy production and storage, and can trigger regulation processes.^108–119^ Small molecule metabolites-based metabolomics have several advantages over the other omics approaches. Genomics may have little impact on expression outcome in the function of a protein, but metabolomics can directly detect the biochemical response to a stimulus.^120–123^ Unlike metabolomics, genomics, transcriptomics and proteomics is unable to dynamically analyze the detailed information of metabolic function in living systems.^124^ Considering time sensitive and accurate phenotypic analysis of live organisms, their individual diagnostic ability is lower than that of metabolomics.^125–129^ As a downstream product of transcriptome, genome and proteome, metabolome includes small molecule metabolites correlate to specific metabolic phenotype and insights into the mechanistic basis and therapeutic targets of diseases (illustrated in Fig. 2). Over the past few years, it has demonstrated significant benefits for discovering biomarkers, disease diagnosis and treatment, and delineating metabolic regulation mechanism.^130–136^ Metabolic signatures from complete system can infer the possible mechanism of diseases and identify therapeutic targets.^137–146^

Metabolome covers a suite of small metabolites with a molecular mass less than 1500 Da, including but not limited to amino acids, lipids, organic acids, and some exogenous chemicals.^40,42,147–151^ All metabolite repertoire is influenced by the physiological activity or exogenous environmental factors.^152–156^ It makes metabolome information data more difficult to interpret.^157,158^ Small metabolites classified as endogenous and exogenous analytes could participate in various metabolic pathways, such as urea cycle, tricarboxylic acid cycle, or fat oxidation.^159–161^ The former includes amino acids such as glycosylation products, histidine and cystine, organic acids such as succinate and citrate, lipids such as glycerolipids and sphingolipids, and other endogenous molecules.^162–166^ A wide variety of biological media has been used from all available body fluids and tissues, including serum, plasma, cerebrospinal fluid, saliva, feces, sweat, tears, urine, breast milk, cervicovaginal secretions.^127,167–171^

Molecular profiling of minor molecules offers invaluable insights into the metabolic function and targets. A disruption of metabolic pathways indicates that metabolomics might be used as a more precise tool for patients when compared with the conventional biomarkers.^126,172,173^ It is vital to understand the biological role of metabolites in regulating biological functions. Numerous strategies have been showed to expand small-molecule metabolites coverage.^174–176^ Mass spectrometry (MS) has been applied to the detection of small molecule metabolites, and allowing interpretation of metabolic changes at the systems-level in health and disease, from whole organisms to single cells.^177–190^ Metabolomics mass spectrometry-based can rapidly discover small molecule metabolites and improve the understanding metabolic mechanism of numerous diseases, and improve the ability for monitoring various metabolic changes in clinical settings.^191–194^ Mass spectrometry coupled with liquid chromatography platforms enhances versatility and sensitivity of identification and quantification of metabolites, precisely facilitates exploration of a large number of small-molecule metabolites from bio-samples, and describes a main picture of general metabolic changes that related to disease alteration.^49,136,195^ Emerging mass spectrometry imaging is a powerful analytical approach for spatial detection, quantification and imaging of endogenous and exogenous molecules.^196–198^ A cross-platform approach by integration of systems biology and small molecule data could discover the regulators of human metabolism into clinical insights.^199–201^ High-throughput metabolic profiling can reveal credible information on the underlying functional metabolic mechanisms.^202–205^

Technique breakthroughs have provided new opportunities to explore metabolic dimensions of diseases. Major analytic techniques for endogenous molecules include nuclear magnetic resonance (NMR) and mass spectrometry. MS can identify the low-abundance metabolites and metabolic alteration along key pathways is identifies by NMR. Recent efforts are directed towards revealing globally spatial distribution of small molecule metabolites and identifying active metabolites beyond their trend analysis and metabolites characterization.^64,206–208^ High-throughput MS imaging (MSI) technology allows for simultaneous visualization of spatial distribution of small metabolite molecules, providing attractive platforms for spatial visualization of metabolic processes to understand the complex communication networks.^209–211^ It is noteworthy that MSI technology has been successfully applied to imaging various human and animal tissues, such as liver, kidney, brain, heart, skin, breast and lens.^212–216^ NMR profiles has been largely used for characterizing biomarker and classified numerous diseases, including kidney diseases, cancer, cardiovascular diseases, Alzheimer’s disease and *etc*.^217–225^ At present, no single analytical method or instrument can fulfill the mission of identification of entire metabolome.^226^ Many reviews have recognized about the combination platform to maximize metabolomics data.^227–229^ Multiple technologies have greatly broadened the level of metabolite coverage, and several reviews have also been widely discussed regarding how different MS and NMR platforms works and their own advantages and disadvantages.^135,230–241^

Small molecule metabolites-based metabolomics can be categorized into targeted and untargeted approaches.^65,75,134,242,243^ Untargeted metabolomics reveals previously unknown metabolic information, and conversely, targeted approach highlight analyzing a set of metabolites, tend to be more sensitive and higher reproducibility relative to untargeted approach.^133,244–249^ Targeted metabolomics tends to analyze a specific known metabolic pathway for the metabolite quantification.^250,251^ However, untargeted metabolomics often focuses on a large number of unknown metabolites without bias and metabolite identification.^252–258^ Untargeted (discovery-based) approach enables global detection of all metabolites that linked phenotype information. Targeted (validated-based) metabolomics focused on the metabolites related to a metabolic pathway of interest. Due to the complex of metabolome, robust data analysis requires the preprocessing raw data followed by multivariate statistical analysis, omics data mining and bioinformatics integration.^259–269^ The larger data sets require the specialized tools for rapid analysis.^270–273^ The progressions such as automatic annotation, in-silico fragmentation and databases construction have advanced to solving these problems.^274–276^ Multivariate statistical techniques are widely applied in mechanistic understanding of metabolic processes, beyond phenotyping and biomarker discovery of various diseases.^277–288^ Data pre-processing software and numerous pattern recognition analysis packages have been reviewed elsewhere.^289–295^ Human Metabolome Database and Kyoto Encyclopedia of Genes and Genomes are the frequently used databases currently in small molecule applications field.^296–301^ Metabolome data can be processed automatically by bioinformatic tools.^302–304^ For instance, MetaboAnalyst tools can generalize network interaction and visualization map derive meaningful biological inferences, which includes numerous modules for pathway analysis and metabolite enrichment analysis with network topology approaches.^305–310^ It can provide a ranked list of potential metabolite biomarkers and fundamental metabolic pathways by allocating small metabolites to relevant biological pathways with pathophysiological basis of disease.

## Identification of bioactive metabolites

Endogenous metabolites are biosynthesized by the host organism or microflora. In 1971, Linus Pauling et al. had used endogenous metabolites to reveal physiological status in biological system. Small molecule metabolites can be produced by catabolism or anabolism, such as peptides, sugars, amino acids, nucleic acids, organic acids, lipids, and fatty acids. Metabolites are the closest link between the genotype and phenotypes, and that reflects the genome, proteome, transcriptome, epigenome, and the interactions with environment.^311^ They play critical roles in biological pathways and serve as valuable bioindicators during cellular processes.^91,312,313^ Metabolic profile could provide a snapshot of complex interplay between environment and intermediary processes.^314–316^ Once specific metabolites to disease pathophysiology are identified and then gain interest in understanding biological biomarkers within mechanistic pathways, using an invasive approach to monitor disease progression and distinguish diseased subjects. To date, metabolic signatures have already been discovered from investigations to uncover biomarkers and gain insight into the ongoing metabolism and treatment targets for numerous diseases.^317–324^

Metabolome are comprehensively characterizing small metabolites in cells, biofluids, organs, or other biological systems. Due to the chemical complexity and dynamic range of the metabolome, the simultaneous identification and reliable quantification of metabolite features are greatly complicated. Biomarker identification can facilitate the diagnosis and prognosis of diseases or individualized treatment, better understanding and exploring potential molecular pathways and mechanisms within disease progression or modulated by drugs. Identification of active metabolite is part of the most important processes in the discovery stage.^325–329^ The biological matrices are complex with thousands of small metabolites in them, the use of analytical profiling techniques identify (global, untargeted, and top-down approach) and quantify (specific, targeted, and bottom-up approach) metabolites contribute to understanding the pathology mechanisms. Given the metabolic profile alterations, the qualitative and quantitative study technique of small metabolite molecules, provides an opportunity for identifying promising biomarkers and predictive model^330–342^.

**Fig. 3:**
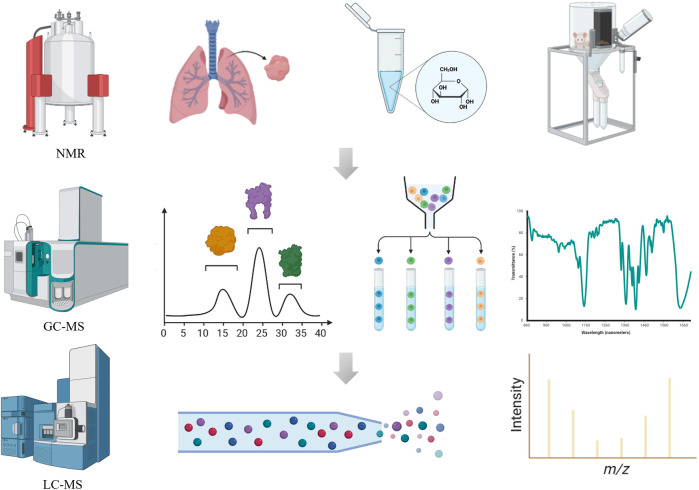
Overview of advanced technology platform for metabolite quantification in biomedicine. Step 1: Sample preparation through deproteinization and/or centrifugation of biofluids. Step 2: Detection of analyte signal through NMR or MS spectroscopy. Step 3: Small metabolites is filtered and quantified for significant biomarkers of interest. The images were obtained using the example data provided by the MetaboAnalyst 5.0 and figures created by BioRender

The identification of the selected minor metabolites can be carried out by a range of analytical technology.^342–344^ Major analytical platforms for small molecules are nuclear magnetic resonance (NMR), liquid chromatography-mass spectrometry (LC-MS), and gas chromatography-mass spectrometry (GC-MS) (Fig. 3). Each technique offers unique advantages in the sensitivity, accuracy, resolution, dynamic range, reproducibility and throughput. A mass spectrometry-based strategy for identifying a list of biological activity metabolites. Moreover, it detects entire metabolites rather than a single metabolite. MS scan, high-precision MS/MS analysis combined with database (e.g., HMDB and METLIN) can provide a large number of the relatively abundant ions and acquire more reliable identifications.^345–347^ It also needs analysis software for highly complex data to complete the metabolite identification and metabolic pathways analysis.^348^ XCMS Online, Open-MS, MZmine and MS-DIAL software are available for peak detection and alignment.^349–351^ Untargeted approach deals with a vast number of unknown molecules and reveals functional changes, and then targeted approach focus on accurate identification and quantitation is subsequent validation including sample preparation, data acquisition and analysis. NMR data processing has been accessible via NMRbox for metabolite identification. Software tools and substantial spectral databases facilitate the identification of small metabolites by both 1D-NMR (e.g., B.I. QUANT, Chenomx NMR Suite, Bayesil, MagMet) and 2D-NMR (e.g., COLMAR).^352–358^ However, combining NMR and MS data greatly improves the metabolome coverage and enhances the accuracy of small metabolite identification, greatly benefit the quality of data.^359^

Numerous groups seek to provide the available online tools for statistical and bioinformatic analysis, e.g., Metlin, MetaboAnalyst, KEGG.^58,360–364^ Small metabolite abundance is quantified depending on peak intensity. The biological activity metabolites are selected by specific statistical cutoff (e.g. a fold changeå 2 or a *p* value < 0.01). Correlations calculated the association between metabolites and clinical features and further evaluated the underlying metabolism differences. To find a panel of metabolites as possible biomarkers for the specific condition, each metabolite needs to be independently analyzed to illustrate the diagnostic ability. The area under the ROC curve (AUC) measured accuracy to see how the metabolites contribute to group separation and ROC analysis could check of the performance of particular metabolites for a diagnostic test.^172,365–368^ To evaluate the overall performance of small metabolites for diagnosis, the sensitivities, AUC values, specificities were evaluated. A total of 24368 metabolites has been published according to recent HMDB 5.0 database.^106^ Number of small metabolites was identified in urine and blood are 5661 and 38,036, respectively. In recent decades, many small metabolites have been discovered in diseases progression, and these studies need emphasize metabolite bioactivity and provide their relevant biological significance.

## Exploring phenotype signatures

Metabolites are end-products or intermediates of the metabolism processes and closely linked to the phenotype of a biological system, which govern the modulating the phenotype function. Level changes of small metabolites could be used as diagnostic and prognostic biomarkers as well as therapeutic targets.^320,369–372^ Metabolome is in constant change, and thus a more reflection of body phenotype than the other “-omics”, such as transcriptomics, proteomics or genomics. Metabolomics tool in clinic measuring variations of metabolites will play a key role for biomarker research, the identification of biochemical pathways involved in the treatment follow-up.^373–377^ Metabolomics obtaining global metabolic profile in biological systems can measure low-molecular-weight metabolites in the biological systems associated with various pathological conditions, could fill gaps between end-phenotypes and genotype.^378–380^

Small metabolites are correlation with the functional status in a biological system. Exploring metabolites and the related metabolic pathways allow a better understanding of how the abnormal metabolism could lead to disease’s onset, and progression.^381–385^ They enter body circulation and then is transferred to target organ and tissues, and then exert a series of biological effects that modulate cell function.^386–390^ Small metabolites could hint proteins acting as modulators of various biological phenotypes and could develop targets for early intervention.^391–395^ Metabolic signatures associated with human phenotype can be identified by various ways including by exploring associations between small metabolites and phenotypes.^396,397^ In addition, research of metabolic signatures has help discovery of potential biomarkers for the diseases. Toward developing effective approaches to evaluate disease progression and therapy responses, a robust and reproducible method is necessary to accurately depict their phenotype. A challenge is to identify the key “signals” of interest in metabolomic data that real influence on phenotype. Identifying molecular signatures that modulate phenotype could be achieved by an appropriate screening way. Mass spectrometry (MS) can detect all the ionizable metabolites without labeling or preselection.^57,255,351,398–403^ High-throughput screening of metabolic signatures that are closest to phenotypes advances to quantify and identify small-molecule metabolites at microenvironments.^404–409^ Single-cell metabolomic methods provide a direct understanding of the phenotypes of cellular activity and environmental changes.^410–414^ High-throughput molecular fingerprints in a wide range of pathological conditions were generated from metabolic profiling of biofluids and have been evaluated neurodegenerative conditions, cardiovascular diseases, metabolic disorders, and various types of cancers.^415–423^

**Fig. 4:**
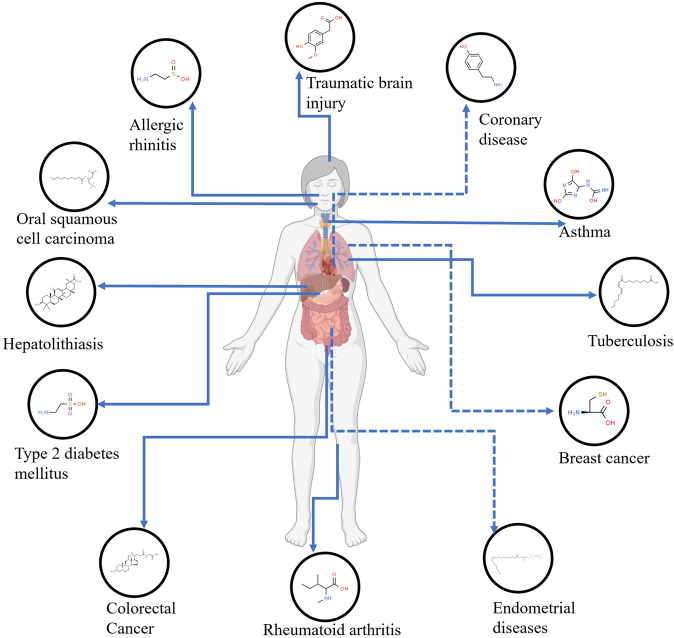
Representative metabolite biomarkers associated with human diseases in clinical studies for disease phenotype, diagnosis, classification, prognosis, and treatment (the detailed information showed in Table 1)

Understanding manifestations of each patient’s metabolic map will allow for precision therapies rather than for the “average patient”. Metabolic profile, a collection of distinct metabolites, could describe human phenotype using small chemical metabolites as index for biochemical traits.^68,424–427^ Because it could reflect a patient’s phenotype, metabolic profile offers a comprehensive, precise and dynamic picture of the phenotype, allows the discovery of small metabolites related with various human phenotypes that link to health, disease or drug monitoring.^100,428–442^ Discrimination between the metabolite profile of diseases could result in potential benefits of identification of early diagnostic or prognostic biomarkers response to predictions.^443–449^ The elucidation of specific metabolic phenotype is essential for identifying potential biomarkers and drug targets, better understanding the underlying pathogenesis during disease progression (Fig. 4). Metabolic phenotyping from biological samples based on the fundamental paradigm of the homeostasis could reflect the substantial changes in the whole metabolism.^450^ The metabolic profiles of patients are dynamic and can be influenced by lifestyle, disease, external or internal stimuli and physiological and pathological condition changes.^451–458^ The biological processes that be related with gender, age, obesity, disease, medication, etc., could change the metabolic profile of an individual.^459–464^ Metabolic profile of biofluid media can directly reflect the particular metabolic status of different tissues or organs, also determine metabolic signatures for identifying the distinct patient subgroups according to disease characteristics.^168,465–472^ Since changes in various pathological conditions can be revealed by metabolic profiles, exploring metabolome could help towards enhancing the disease diagnosis, prognosis, surveillance, and personalized treatments. Metabolic changes serving as biomarkers for early diagnosis and potential therapeutic target, play significant pathological effects on regulated metabolism.

Several small metabolites analyses have been carried out to identify the specific metabolic phenotype profile relevant with disease progression and characterize alterations of metabolic signatures which may be used as potential biomarkers in clinic. The study by Liu et al. was aimed at characterizing the distinctive inflammatory phenotypes, and then identify the metabolic signatures and pathways. It demonstrated adenosine 5'-monophosphate, allantoin and nicotinamide correlate with metabolic changes to predict asthma inflammatory phenotypes.^473^ Another study has reported that the differential metabolites including glycerophosphocholine, rosterone sulfate, and elaidic carnitine as potential indicators can predict abortion rate of polycystic ovary syndrome, with an AUC of 0.933, 0.941, 0.933 for high predictive performance, respectively.^474^ LC-tandem MS was performed to characterize the serum metabolic signatures of hepatolithiasis, and identified 277 metabolites, AUC values for metabolites including18-β-glycyrrhetinic acid and PC (4:0/16:2) were up to 0.90, may have clinical value for hepatolithiasis.^475^ A study showed that polyunsaturated fatty acids and bile acids as potent markers were closely related to the severity and chronicity of drug-induced liver injury patients, respectively.^476^ In addition, the distinct metabolic signatures at the acute phase of COVID-19 patients compared to the recovery period, suggesting arginine and tryptophan metabolism as main pathways with a probable link to disease severity.^477^ In a quantitative profiling study that focused on urinary metabolic signatures including homovanillate, L-methionine, and thymine as indicators of traumatic brain injury.^478^ Concerning metabolic signatures of tumor growth stage, such as colorectal cancer, sporadic colorectal adenoma, and the potential metabolites such as D-mannose, sarcosine, 4,5-trimethoxybenzoic acid are found by serum metabolic screening.^479^ By metabolic features of PCa analyses, Yu et al. discovered a series of altered metabolites that were related to TCA, glycine cleavage system, fatty acid metabolism. Importantly, Glu/Gln had high predictive power when detecting PCa patients (AUC = 0.984), with a higher sensitivity (96.6%) than PSA (94.4%).^480^ In summary, these studies show the small molecule phenotype signatures offers new avenues for better understanding biological metabolic processes of diseases, and for developing new biomarkers to improve patient management in clinic.

## Promising biomarkers

According to NIH Biomarkers Definitions Group, biomarkers were defined as features which are measured as an index or sign for physiological, biological, pathological, or pharmacological processes. Biomarker have characteristics that can be quantified, analyzed and associated with human phenotype and could be used for early disease detection, improve outcomes of treatments and selection of therapeutic strategy, and reduce disease-related mortalities, and lead to the identification of the therapeutic targets. Although the extensive efforts, currently used biomarkers in clinic are lacking adequate sensitivity and specificity for disease early detection and treatment monitoring. A growing number of biomarkers in urine, blood, plasma or saliva, have been considered to identify intermediate phenotypes with a clearer picture for predicting the response to therapy. Altered metabolisms have recognized as biomarkers. Metabolic profile can describe the underlying molecular picture of disease disorder or phenotype. Therefore, to improve the patient management, more precise biomarkers in biofluids are needed. Discovery of metabolic biomarkers will improve patient pretreatment and response to therapy.

Recently, a variety of biomarkers were discovered and employed to detect early-stage disease and predict disease progression, clinical outcome or drug response (Table 1). It can be a group of metabolites, a metabolite, or a molecular feature. The presence of a disease suggests the metabolite concentration has abnormal change (lower or higher concentration) is a sign of a perturbed or dysfunctional metabolic pathways of systemic homeostasis. There are huge advantages to consider and apply metabolic information during discovery phase that focusing on the understanding of the biological system associated with the metabolic pathways and can provide novel biomarkers and targets. Thus, unlike proteins and genes, metabolites as signatures of biochemical activity are closely correlate with human phenotype, since they play a key role in cellular signaling regulation and physiological function control. Therefore, discovery of altered metabolic features related to phenotypic variation produced insights into pathophysiology, mechanistic basis and therapeutic targets of metabolic diseases.

**Table 1:** Small-molecule metabolites and relevant metabolic alterations associated with human diseases in recent clinical studies

Disease type	Reference	No. of patient	Biological matrix	Research aim	Analytical platform	Potential biomarkers	Key pathways
Hepatolithiasis	Cong Wang et al.^475^	30	Serum	Phenotype	UPLC-MS	18-β-Glycyrrhetinic acid, PC (4:0/16:2)	Taurine and hypotaurine metabolism, bile secretion
Traumatic brain injury	Elani A. Bykowski et al.^478^	8	Urine	Phenotype	NMR	Homovanillate, L-methionine, thymine	Purine metabolism
COVID-19	Laura Ansone et al.^477^	32	Serum	Phenotype	LC-MS	L-phenylalanine, tyrosine	Tryptophan and arginine metabolism
Asthma	Ying Liu et al.^473^	119	Sputum	Phenotype	UPLC-MS	Adenosine 5′-monophosphate, allantoin, nicotinamide	Histidine metabolism, nicotinate, nicotinamide metabolism
Diabetic kidney disease	Shijia Liu et al.^526^	1513	Serum	diagnosis	GC-MS	Glycerol-3-galactoside	Galactose metabolism, glycerolipid metabolism
Nonalcoholic fatty liver disease	Xuemei Wang et al.^519^	149	Serum	diagnosis	UPLC-MS	Theophylline, 2-hydroxyphenylacetic acid, lysophosphatidylcholine (24:1(15Z))	Caffeine metabolism, choline metabolism and sphingolipid metabolism
Tuberculosis	Xin Hu et al.^520^	64	Plasma	diagnosis	UPLC-MS	9-OxoODE, DL-Norvaline, Ethyl 3-hydroxybutyrate	Lipid synthesis and biosynthesis of glutathione
Breast cancer	Rui An et al.^521^	216	Plasma	diagnosis	UPLC-MS	Sphingomyelins, glutamate, cysteine	Alanine, aspartate and glutamate pathways, glutamine metabolic pathway
Oral squamous cell carcinoma	Xibo Li et al.^524^	72	Plasma	diagnosis	UPLC-MS	Decanoylcarnitine, cysteine, cholic acid	Cholic and amino acid metabolism
Salivary gland tumors	Mengmeng Wu et al.^544^	30	Serum	Classification	UPLC-MS	Serine, lactic acid	Metabolism of fats and fatty acids, anaerobic glycolysis
Endometrial diseases	Xingxu Yan et al.^552^	326	Serum	Classification	UPLC-MS	6-ketoPGF1α, PA(37:4), LysoPC(20:1)	Glycerophospholipid metabolism
Papillary thyroid cancer	Shuang Yu et al.^550^	148	Plasma	Classification	UPLC-MS	Capryloylglycine, valeric acid, triethanolamine, imidazoleacetic acid, etc.	
COVID-19	AlbertoValdés et al.^551^	145	Plasma	Classification	HPLC-MS	3-Hydroxibutirate, linoleic acid, LPC (14:0 and 18:2), LPE (22:6), kynurenic acid	Metabolism of carnitines, ketone bodies, fatty acids, and lysophosphatidylcholines
Bladder Cancer, Prostate Cancer, Renal Cell Carcinoma	Sujin Lee et al.^554^	24, 29, 12	Urine	Classification	NMR	4-Hydroxybenzoate, N-methylhydantoin,creatinine, glutamine, acetate	
Coronary atherosclerotic heart disease	Yuxuan Fan et al.^547^	60	Serum	Classification	UPLC-MS	5-Cholesten-3β, N-Acetyl-lysine, tyramine, biliverdin, urocanate, etc.	Energy, lipid m and amino acid metabolism
COVID-19	IvaylaRoberts et al.^577^	120	Serum	Prognosis	UPLC-MS	Deoxycytidine, ureidopropionate, kynurenine, multiple short chain acylcarnitines	Pyrimidine, kynurenine, and energy metabolism
Type 2 diabetes mellitus	Julia Brunmair et al.^580^	8	Sweat, tear fuid	Prognosis	HPLC-MS	Nicotinic acid, taurine	
Acute ischemic stroke	Chaofu Ke et al.^579^	143	Blood	Prognosis	HPLC-MS	LysoPC(18:1), Lys Val Phe Lys, LysoPC(18:2), PS(O-18:0/0:0)	
COVID-19	Victòria Ceperuelo-Mallafre et al.^575^	273	Serum	Prognosis	GC-MS	Pyruvate, lactate, succinate, a-ketoglutarate	Krebs cycle
COVID-19	Lucas Barbosa Oliveira et al.^537^	242	Plasma	Prognosis	HESI-Q-Orbitrap	Cholesteryl ester CE (18:3)	Glycerophospholipid and porphyrin metabolism
Neuroblastoma	Sebastiano Barco et al.^578^	172	Plasma	Prognosis	HRMS	3-O-methyldopa	
Colorectal Cancer	Xinyi Shen et al.^581^	197	Tissues	Prognosis	UPLC-MS, HPLC-MS	Asparagine, serine	Asparagine synthesis pathway, serine metabolism
Allergic rhinitis	Rui-Li Yu et al.^619^	43	Serum	Treatment	GC-MS	Hypotaurine, taurine, L-alanine,	Taurine and hypotaurine metabolism, and alanine metabolism
Epileptic spasms	Jingya Yan et al.^621^	34	Cerebrospinal fluid	Treatment	LC-MS	Kynurenic acid, 3-hydroxykynurenine, xanthurenic acid, anthranilic acid, quinolinic acid, picolinic acid, etc.	Kynurenine pathway
Rheumatoid arthritis	Matthew R. Medcalf et al.^624^	20	Plasma	Treatment	GC-MS	N-methylisoleucine, 2,3-dihydroxybutanoic acid	
Schizophrenia	Xiaoni Guan et al.^622^	25	Plasma	Treatment	UPLC-MS	Methyl n-formylanthranilate	Kynurenine pathway of tryptophan metabolism
Psoriasis vulgaris	Dan Dai et al.^623^	88	Blood	Treatment	UPLC-MS	SM (d16: 0/17:1), SM (d19:1/20:0), Cer (d18:1/18:0), PC (18:0/22:4), PC (20:0/22:4)	Lipid metabolism dysfunction
Polypoidal choroidal vasculopathy	Yinchen Shen et al.^620^	93	Serum	Treatment	UPLC-MS	Dacylglycerophosphocholines, lysophosphatidylcholine, fatty acids, phosphocholine	Lysophosphatidylcholine and diacylglycerophosphocholine metabolism
Breast cancer	Ehsan Irajizad et al.^617^	88	Plasma	Treatment	UPLC-MS	Polyamines	
Bladder Cancer	Juntao Zhuang et al.^618^	18	Serum	Treatment	NMR, UPLC-MS	Glutamine, glutamate, hypoxanthine	Amino acid pathways
Colorectal cancer	Yu Yuan et al.^676^	30	Serum	Function	UPLC-MS	Glycodeoxycholic acid	Poly (ADP-ribose) polymerase-1
Breast cancer	Yi Xiao et al.^22^	330	Tissues	Function	LC-MS	Sphingosine-1-phosphate	Ceramide pathway
Acute traumatic brain injury	Ilias Thomas et al.^673^	716	Serum	Function	UPLC-MS	Lysophosphatidylcholines, ether phosphatidylcholines, sphingomyelins	
Type 2 diabetes	Maria Giovanna Scarale et al.^675^	279	Serum	Function	LC-MS	Hexanoylcarnitine, kynurenine, tryptophan	
Kawasaki disease	Qiongjun Zhu et al.^674^	79	Blood	Function	LC-MS	Palmitic acid	Generation of reactive oxygen species
Aortic aneurysm	Hongtu Cui et al.^164^	1705	Plasma	Function	UPLC-MS	Succinate	p38a/CREB/OGDH axis

*UPLC-MS* ultra performance liquid chromatography-tandem mass spectrometry, *NMR* nuclear magnetic resonance, *LC-MS* liquid chromatography-tandem mass spectrometry, *GC-MS* gas chromatography coupled to mass spectrometry

Given metabolism plays fundamental roles in characteristic metabolic alterations to gain deep insights into disease pathogenesis, small metabolites could emerge as potential targets for developing predictive biomarkers, and therapeutic targets. The precision treatment of metabolic disorders remains a huge challenge due to the imprecise diagnosis and involved incomplete understanding of pathophysiological process. To practice precision treatment, it is necessary to investigate small biomarkers that carefully consider phenotype determination. To establishing quantitative fingerprint and detection of endogenous metabolite biomarkers in easily obtainable and less intrusive biofluid may help to establish the close relationship between disease process and metabolic changes that contribute to body dysfunction of mechanistic basis of metabolic diseases. Currently, it is a challenge to rapidly detect disease using specific metabolite signatures at initial stages. Despite many biomarkers have been discovered in clinic, other biomarkers have not undergone their clinical validity and usefulness, preventing them advanced into clinical treatment. Advanced technology has greatly facilitated the discovery of biomarkers insights into metabolic regulatory and signaling activities that are strongly associated with human phenotype. Furthermore, biomarkers for the prediction, prognosis, and monitoring therapy, after the biomarker discovery phase, need GC or LC-MS, and NMR spectroscopy analytical techniques. Advanced analytical techniques could insight into the concentration detection of potential metabolite biomarkers within its early stages. Advanced platforms, especially using LC/MS/MS, facilitate detection, quantification, and characterization of small metabolic molecules (e.g., peptides, carbohydrates, amino acids, and fatty acids) involved in metabolic and catabolic processes, and greatly enhanced their translational capability.

Some representative potential metabolite biomarkers are currently screened (Fig. 4). A six-metabolite panel (beta-alanine, homoserine, 3-hydroxykynurenine, aspartate, tyrosine and ornithine) was quantified as potential blood-based biomarkers, and considers as a potential diagnostic or prognostic assay for Parkinson’s disease.^481^ Eva et al. had profiled serum metabolite signatures in early breast cancer participants and found that circulating metabolites: glutamine, tyrosine, proline, histidine, alanine and citrate can significantly correlate with tumor proliferation.^482^ Interestingly, a panel of two potential predictive metabolites (palmitic amide and deoxycholic acid) in serum was reported as potential biomarker of Crohn’s disease patients, and its metabolic disturbance involved the fatty acids, bile acid biosynthesis, and energy metabolism.^483^ Additionally with the use of correlation analysis and ROC curve analysis, the characteristic metabolites including alanine, glucose, lactate, glycine and threonine were identified in pulmonary arterial hypertension patients, and threonine and lactate were markedly correlated with pulmonary vascular resistance and arterial pressure.^484^

In a study that focused on biomarkers associated with gouty arthritis progression in patients, serum metabolic profiles were screened N1-Methyl-2-pyridone-5-carboxamide, kynurenic acid, 5-and hydroxyindole acetic acid.^485^ A multi-omics model with machine learning approaches was developed for discovering metabolite biomarkers predicting COVID-19 patients.^486^ Interestingly, 5-oxoproline can be used as a key biomarker for acute ischemic stroke.^487^ Metabolic profiling model based on seven metabolite candidates in plasma samples can provide powerful early survival prediction capabilities for ST-segment elevation myocardial infarction patients.^488^ Potential small metabolites included LysoPC(15:0), docosapentaenoic acid, propionyl carnitine, LysoPC(14:0), and phenylalanine were constructed a risk score for dose-response relationship with metabolism abnormalities and metabolic syndrome.^381^ Plasma metabolic profiling revealed four circulating metabolites (glutamate, pseudouridine, N-acetyltryptophan and leucylleucine) were identified in diabetic retinopathy patients.^489^ It has been reported that candidate biomarkers arachidonic acid and 13(S)-HODE associated with Akt pathway were potential biomarkers of non-small-cell lung cancer.^490^

## Diagnostic biomarkers

Early diagnosis and effective prevention are of great importance and has attracted great attention for improving treatment and new therapeutic targets. For ideal biomarkers, molecular compound should be readily measurable in invasive biological media. Given metabolites are downstream expression of genome, closely indicate phenotypic fingerprints at a particular physiological period.^491–494^ One of the major advantages of metabolome over genome is that it can reflect environmental impact and provide global photograph of individual pathological conditions at any time point. Timely diagnosis is crucial, and the screening of small metabolites could play pivotal role in disease diagnosis. Therefore, the need for prompt diagnosis indicates the huge potential of advanced methods that reflect phenotype and therefore function changes. Since small metabolites indicate end-products of physiological processes, exploring whole metabolome can better understand disease pathology and mechanisms of intervention.^495–498^

**Fig. 5:**
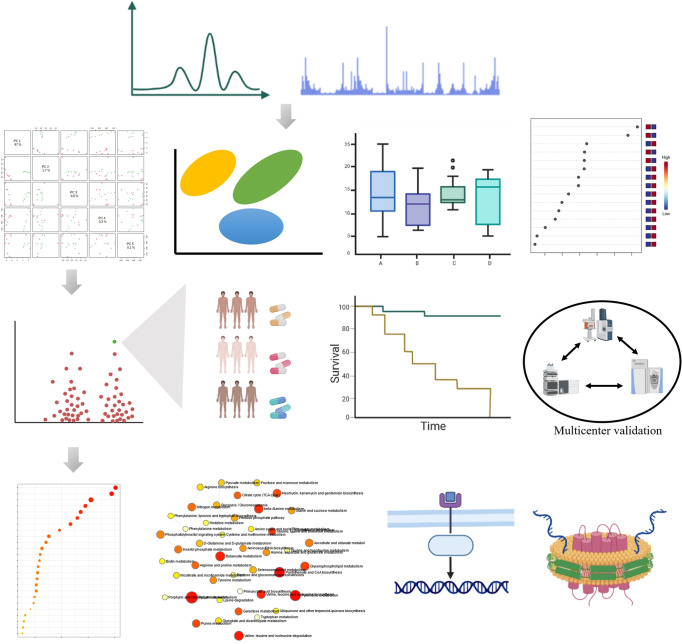
Potential roles and applications of small-molecule candidate metabolites for biomarker discovery, diseases diagnosis, prognosis, and monitoring treatments in biomedicine. Compound detection, metabolites are detected by using specific detection techniques; data pre-processing, raw signals are then pre-processed to produce data in a suitable format for subsequent statistical analysis; then, data normalization is used to reduce the system and technical bias; data processing, for untargeted studies, metabolites are identified from spectral information in some given database; statistical analyses, univariate and multivariate statistical analyses are used to identify significantly expressed metabolites; biomarker discovery from multicenter, the discriminant metabolites originated from metabolomics approaches may become promising candidate molecules to aid disease diagnosis, and risk stratification; function analyses, next, the significantly expressed metabolites are subsequently linked to the biological context by using enrichment and pathway analysis. The images were obtained using the example data provided by the MetaboAnalyst 5.0 and figures created by BioRender

Advanced analytical technology for small metabolites profiling features in distinguishing or determining disease pathophysiology associated with disease subtypes, progression, and treatment. Disease detection techniques over traditional methods are necessary for initial diagnosis, and also provide an effective approach to screen the right populations, assess drug efficacy, guide the choice of treatment or track disease progression, provide better patient care. Rapid progress in omics by high-throughput technology including LC-MS, GC-MS, and NMR, focused on characterization of metabolic phenotype, has allowed for simultaneous determination of a large number of small metabolic products in biological specimens.^499–503^ Omics approaches for biomarker discovery of early disease diagnosis could be achieved by analytical tools together with pattern recognition analysis (Fig. 5). Typical examples of these approaches consist of metabolic profiling, metabolic footprinting, metabolic fingerprinting, flux and target analysis, each of which has played a significant effect in clarifying the related metabolic pathways, understanding disease mechanisms and pathological processes.^504–508^ It can accurately detect the changes in distinguished features of metabolism, remains indispensable for disease detection.

**Fig. 6:**
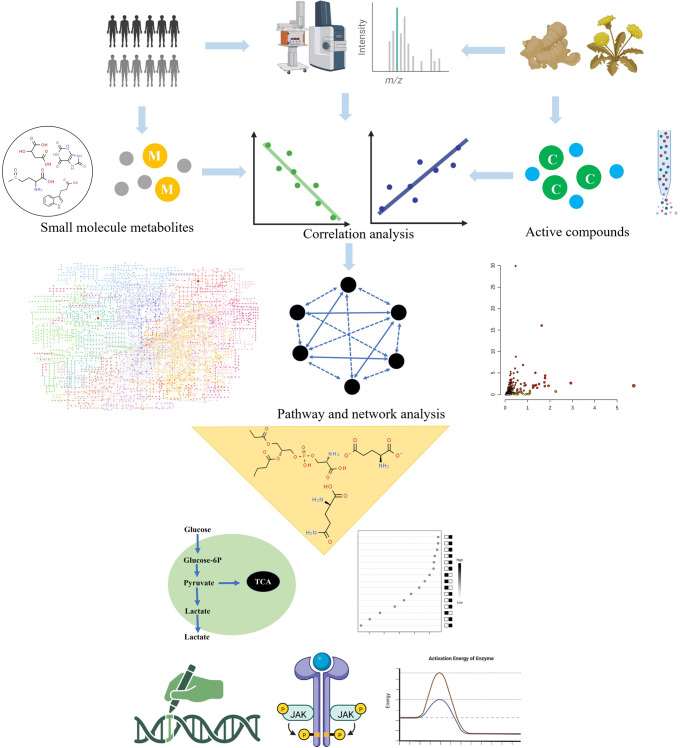
Schematic diagram of an integrated pharmacology framework for discovery of bioactivity-correlated constituents, target identification and action mechanism of herbal medicine and natural products. The first stage discovers active compounds of treatment-related herbs followed by construction of correlation analysis network of treatment-related herb-compound and small molecule metabolite (Correlations based on the abundance scored value). Next is that highlight the main active constituents from identification of new candidates from natural products, and then elucidate the underlying mechanisms by target virtual screening and identification, until the final step of in vitro and in vivo tests. The images were obtained using the example data provided by the MetaboAnalyst 5.0 and figures created by BioRender

The metabolites linking between genotype and phenotype will result in biomarker identification for the early diagnosis, detection, and response to treatment, better understanding the complex disease pathophysiology that dramatic functional changes. Metabolic signature of disease could assess the risk or earlier diagnosis, detection, treatment monitoring, specific disease subtypes, and help selection of targeted treatment to match metabolic alterations of diseases related to phenotypic variation.^509–514^ To identify metabolite profile changes in early diagnosis stage of diseases is important for improving the prognosis, treatment and management. A larger number of single metabolite or a panel of the dysregulated metabolites can build diagnostic models that hold diagnostic power and are capable of differentiating patients.^515–518^ Small molecule metabolites reflecting dynamic pathological information is gradually moving towards clinical practice, and has been proven accurate enough for satisfactory diagnostic performance to predict diseases or early diagnosis or discriminate patients. Figure 6 shows how small metabolites could build metabolic blueprint of predictors in identifying biomarkers for early complex disease detection. Pathway analysis could expound altered metabolic alteration and show disease treatment options. Its application in all aspects of diagnostic potential has been proved in the research of metabolic disorders involved in disease progression, such as diabetes, metabolic syndrome and obesity.

Regarding establishing early clinical diagnostic tool, a study was performed to identify differential and functional metabolites of early NAFLD. New candidates were discovered, including the upregulated theophylline and 1-naphthylmethanol, downregulated lysophosphatidylcholine (24:1(15Z)) and 2-hydroxyphenylacetic acid. It can achieve a high diagnostic power in the discovery phase (80.99%) and validation phase (75.23%).^519^ Study carried out by Xin et al. highlighted how metabolite biomarkers and metabolic profile can serve as biomarkers for precision diagnosis of various types of tuberculosis.^520^ Further, it also demonstrated that potential of machine learning method combining metabolome in screening out diagnostic biomarkers from big data set. Parallel study has been carried out in plasma samples focusing on the metabolic characterization of breast cancer patient, and revealed specific metabolic profiles, identified a panel of glutamate, sphingomyelins, and cysteine that showed high predictability that can be used as diagnostic biomarkers.^521^ Previous study reported LPC (18:2/0:0) level correlate with diastolic dysfunction and glycyl tyrosine correlate with reduced lower left ventricular ejection fraction, indicating they can detect cardiovascular risk.^522^ An integrated multi-platform analyses were used to screen biologically significant metabolites linked to Esophageal squamous cell carcinoma patients.^523^ It found the close link lipid, amino acid metabolism, and a diagnosis panel of citrulline, l-carnitine, acetyl-carnitine, tryptophan and lysine selected as potential biomarkers in distinguishing patients. Metabolic pathway analysis obtained biomarkers associated with oral squamous cell carcinoma that closely related to amino acid and cholic acid metabolism. Further, a diagnostic panel was established and constituted of cysteine, cholic acid, decanoylcarnitine, and had high early diagnosis power (AUC = 0.998).^524^ According to Lunyera et al., urine tricarboxylic acid cycle signatures are potential indicators at early-stage diabetic kidney disease progression.^525^ It has proved that glycerolipid metabolism and galactose metabolism are the main metabolic pathways, and serum metabolite glycerol-3-galactoside can be used as an independent indicator to predict diabetic kidney disease.^526^ Here, these instances of clinical trials based on endogenous small molecule metabolites expand the coverage of metabolic biomarkers for disease diagnosis.

## Disease classification and stratification

Clinicians need rapidly assess disease stratification risk and with adequate accuracy. Recently, omics combination approach has employed as a promising strategy for generating information on detecting early metabolic alterations which could contribute to the disease classification, stratification and progression for diseases that are immediately associated to biologically meaningful metabolism, such as cardiovascular diseases, cancer, diabetes, and obesity.^527–530^ The right choice of small molecule metabolites that correlate with pathological states can help making decision and lower-costs from the pilot testing into the clinic. Metabolic profiles of diseases are able to characterize disease signatures for discovering and identifying diagnostic biomarkers and many unexpected mechanistic pathways that involved in disease pathogenesis. Classically, endogenous small molecules metabolite screening combined with the traditional risk assessments enable characterization of metabolic phenotypes even before manifestation of symptoms and have the potential to improve non-invasive diagnostics and disease classification with great potential to translate them into clinical settings.^531–535^ According to small molecule metabolite profiles or fingerprints shown ability to predict disease risks, the big data being collected on artificial intelligence or big data mining will contribute to disease stratification analysis as an integrative tool that assists clinicians in making decisions.^536–538^ Over the past few years, one of the most striking aspects of screening of endogenous small molecules metabolite of systemic metabolome alterations particularly has evolved to gain a much broader dimension, also showed great potential for differentiating disease subtypes.

Importantly, a large number of cohort studies have been carried out to help establish a more effective and reliable risk performance model for disease stratified risk events. In pancreatic ductal adenocarcinoma, a panel of three small metabolites including creatine, proline, and palmitic acid can exhibit a beneficial performance for distinguishing pancreatic ductal adenocarcinoma from benign pancreatic neoplasms or healthy controls.^539^ A study has focused on characterizing the metabolic subtypes of pancreatic ductal adenocarcinoma and analyzing the relationship between long-term prognosis and metabolic subtype.^540^ It did not reveal the metabolic differences at the clinical stages and choline-like type showed better prognosis among metabolic subtypes. Interestingly, a metabolites biomarker panel can precisely predict the overall survival of pancreatic cancer and distinguish tumors from normal pancreatic tissues in a clinical setting.^541^ A recent study investigating potential biomarkers for screening and diagnosis of lung metastases, some low-molecular metabolites such as indoleacrylic acid, L-tyrosine, retinol, L-octanoylcarnitine and decanoylcarnitine were selected and found they had high AUCs values and showed a strong ability to differentiate between pulmonary metastatic carcinoma and other subtypes.^542^ An integrated metabolome and lipidome platform discovered four differential metabolites including D-glyceric acid, cortisol, 2-(methylthio) ethanol, N-acetylhistamine and then established a differentiation model for precise pathological classification of squamous carcinoma and non-small cell lung adenocarcinoma.^543^

Serum metabolic profiles in salivary gland tumors patients were investigated to gain a better understanding of the disease risk stratification. A total of 32 small metabolites were identified, and a risk predicting model based on the gradually upregulated serine and lactic acid was developed in benign and malignant stages.^544^ In medulloblastoma, a panel of two urine metabolites including cortolone and tetrahydrocortisone showed a high accuracy for diagnosis and monitoring.^545^ A machine learning-derived nomogram models using thiamine triphosphate, diabetes duration, and systolic blood pressure were established for early diagnosis and accurate prediction of diabetic retinopathy.^546^ Metabolic alterations in amino acid, energy, lipid, and metabolism could distinguish the different stable and unstable types of coronary atherosclerotic heart disease.^547^ Moreau et al. analyzed salivary metabolome in primary burning mouth syndrome and found tyrosine pathway (L-tyrosine, tyramine, L-dopa) can differ patients according to the levels of pain.^548^ A study performed by tissue-based spatial metabolomics with mass resolution imaging had developed classification system of gastric cancer subtypes and insight into their distinct metabolic pathways and molecular characteristics.^549^

A metabolic biomarker panel was discovered in discovery cohort to discriminate papillary thyroid cancer from benign thyroid nodule with 91.89% sensitivity, AUC of 97.03%, and 92.63% specificity, and in validation cohort displayed 86.57% sensitivity, AUC of 92.72%, and 92.50% specificity, and can improve stratification of thyroid microcarcinoma.^550^ In recent work, several metabolites such as carnitines, fatty acids, ketone bodies, bile acids, purines and tryptophan, were obtained as early biomarkers to distinguish from the early-stage and end-stage coronavirus disease 2019.^551^ Using a ROC curve and logistic regression analysis, a biomarker panel of PA(37:4), 6-keto-PGF1α, PS(36:0), and LysoPC(20:1) demonstrated good classification and diagnostic ability in distinguishing endometrial polyps from endometrial hyperplasia or endometrial cancer in the validation set.^552^ Notably, targeted metabolomics analyses identified gamma-aminobutyric acid markedly reduced in COVID-19 patients and its change levels with high sensitivity that allowed for COVID-19 stratification.^553^

Urinary metabolic features of prostate cancer, bladder cancer, and renal cell carcinoma have been carried out to determine and reveal that *N*-methylhydantoin, 4-hydroxybenzoate, creatinine, acetate and glutamine had significantly discriminatory accuracy among groups.^554^ When the level of a specific antigen is located in the range of 4–10 ng/ml, differential metabolites are screened to effectively distinguish between benign prostatic hyperplasia and prostate cancer.^555^ As for the metabolic perturbations in vivo, Alotaibi and colleagues followed the bioactive lipid molecules screening approach and reported the 5 metabolites as biomarkers of disease severity differed between pulmonary artery hypertension with systemic sclerosis and idiopathic pulmonary arterial hypertension, and provide important underlying mechanistic basis in subgroups of pulmonary artery hypertension.^556^ Furthermore, Luo et al. performed a comprehensive analysis of metabolome data, and relevant metabolite dehydrophytosphingosine and 9-cis-retinoic acid had proved to be the most discriminative biomarkers for ventricular fibrillation phenotype, had the high predictive probability based on their combination model.^557^ Albillos et al. conducted metabolome with multivariate analysis to examine potential biomarkers such as acyl-carnitines, bilirubin, tyramine, for differentiation between Parkinson’s disease and essential tremor.^558^ These studies show a great potential for screening biomarkers for better disease stratification to advance the understanding pathophysiology, allowing therapeutic options.

## Prognosis biomarkers

The lack of symptoms in the prognosis stages makes early disease diagnosis difficult. Prognosis biomarkers are important in order to reduce complex disease mortality. It is essential to identify prognostic biomarkers that could facilitate decision making by clinicians and promote individual therapy. Identification of useful prognosis biomarkers remains a huge challenge in clinic. However, regular tests offer low specificity and sensitivity, leading to inadequate early-stage diagnosis or risk assessment. To improve the risk stratification and prevention of disease, benefit from therapy, it need insight into multiple prognosis biomarkers and simultaneously quantify in a high-throughput way. Interestingly, a great advantage of small metabolites as biomarkers likely occurs a panel of multiple metabolites with markedly concentration changes correlated with disease status.^559–562^ Interestingly, one of the significant advantages of metabolite biomarkers may be that they are composed of multiple metabolites, and their concentration changes are significantly related to disease status.^563–567^ The response to drug therapy can accurately monitor the changes of small metabolites in biological media (e.g., urine and blood).

Large amounts of studies have analyzed the metabolic profiles of patients to identify potential small biomarkers with prognosis utility in the clinic (Table 1). These researches include huge efforts to develop simple, inexpensive, and novel diagnostic applications, to enhance knowledge on the predictive or prognosis biomarkers of the diseases and its complications. Metabolic deregulation could affect various molecular biological processes (e.g., cell apoptosis or invasion) that contribute to disease progression and impact patient survival. Of interest to physician is the great potential of small molecule metabolites an invaluable tool from a prognostic point of view. Instead of a single biomarker, multiple small metabolites corresponding to particular phenotypes are anticipated to yield a higher selectivity and sensitivity.^568–570^ Both un-targeted and targeted approach have also been conducted identifying specific metabolites or predictor biomarkers which were linked to metabolic alterations.^571–574^

Analyses of the relative level of tricarboxylic acid by semi-targeted serum metabolomics shows that circulating pyruvate is an effective prognostic biomarker of COVID-19, which means that the quantification of pyruvate is a clinical support for prognosis prediction.^575^ Metabolic profiling of plasma reveals COVID-19 affected porphyrin and glycerophospholipid metabolism, respectively.^576^ Small metabolites in porphyrin and purine pathways were markedly elevated in severe group, indicating that they can be used for prognostic biomarkers. Prognostic tests based on intermediary metabolites such as ureidopropionate, deoxycytidine and kynurenine could improve COVID-19 patient treatment outcome and severity.^577^ One example comes from Barco et al., who used a targeted metabolite profiling approach to discover the high expression of 3-O-methyldopa was associated with worse prognosis in neuroblastoma patients.^578^ A study performed untargeted metabolomics had revealed eight metabolic biomarkers were identified as prognostic biomarkers of acute ischemic stroke.^579^ As an example according to Brunmair et al., metabolic phenotyping of tear fluid has been successfully established and revealed taurine and nicotinic acid represent new biomarkers were elevated in the diabetic cohort, and supports prediction of disease development.^580^ Furthermore, metabolite profiles also showed that asparagine synthesis was increased and associated to poor prognosis for female colorectal cancer patients.^581^

## Metabolic pathways

Aberrant metabolism is a necessary pillar as a hallmark of disease, e.g. lactate, pyruvate metabolites can assist in cellular proliferation. Comprehensive understanding and investigating mechanistic pathways can provide powerful evidence for precise diagnosis, phenotypic classification, prognosis and treatment of patients. Metabolic pathway analysis can be performed with benefiting mechanistic explanations of therapeutic targets for metabolism-related diseases.^582–584^ The altered metabolites are significantly correlated with metabolic pathways and biological processes involved in the disease progression. In addition, the differential metabolites are likely to be one of the most important information to explain the pathogenesis mechanism. From a metabolic perspective, small molecule metabolites whose altered concentrations could reflect phenotypes and elucidate pathophysiological changes of complex diseases, provides clues regarding alteration of metabolism in dysfunction, helps functional interpretation of metabolic perturbations in vivo related to phenotypic variation.^585–588^ In this context, small molecule metabolites are associated with diagnosis or prognosis in metabolic processes and alteration in treatment of systemic homeostasis. Targeting metabolic pathways can regulate the abnormal metabolisms and finally alleviate disease syndromes.

Small molecule metabolites associated with specific metabolic phenotype can be used to screen early disease symptoms and monitoring its progression, through measuring endogenous metabolite alterations in biofluids or tissues.^589–591^ Discovery of small metabolite by high-throughput, non-invasive, and cost-effective metabolomics are quite useful to compute metabolic pathways that link complex chemical reactions involved in the biological process. Advanced metabolomics technology could amplify the small changes of differential metabolite expression to achieve a wide coverage and then reflect functional changes, deeply reveal action mechanism.^592,593^ This approach enables providing the key information for further exploration of metabolic signatures and potential biomarkers, mechanistic in-depth understanding, and therapeutic targets for treatment. Metabolite can be used as an early indicator of pathological changes prior to development of disease symptom. Several available software platforms have been designed to facilitate metabolic pathway analysis for small molecule metabolites.^594–596^ Particularly, Ingenuity Pathway Analysis and MetaboAnalyst could be used to clarify the relevant metabolic pathway network change associated with small molecule metabolites found in omics data, enable integration for biological interpretations.^58,360,597–599^ The online databases, such as KEGG, provide huge information about a large number of biological pathways and can be easily used to determine and visualize the metabolic pathways and metabolite interaction network involved in fundamental biological processes. These comprehensive tools to the biological interpretation help the identification of differentially altered analytes and dysregulations of pathways.

Previous report had shown that metabolic alterations in clinical hypothyroidism and subclinical hypothyroidism linked to various potential metabolite biomarkers suggesting that impacting onsteroid hormone biosynthesis, primary bile acid biosynthesis, lysine degradation, purine metabolism and tryptophan metabolism.^600^ Recently, Marino et al. performed multivariate network analysis to identify the core pathways in the *advanced* stage of Amyotrophic lateral sclerosis, and suggested the metabolic alteration of lysophosphatidylcholine, sphingomyelin, and phosphocholine metabolism, consistent with repairing inflammation and neuronal degeneration.^601^ Metabolic dysfunction in glycerophospholipid metabolism, arginine and proline metabolism, and tryptophan biosynthesis of invasive ductal carcinoma patients was also observed by pathway enrichment analysis.^602^ Alterations to metabolic pathways included glycerophospholipid metabolism, D-glutamine and D-glutamate metabolism associated with atrial fibrillation have been broadly explored at small metabolites level. A study has focused on characterizing the specific and precise metabolic features of atrial fibrillation subtypes, indicated that small-molecule metabolites may facilitate effective treatment.^603^ Additionally with the use of untargeted metabolomics, a study demonstrated serum biomarkers of progression of diabetic retinopathy in Asians, and there were 171 metabolic features including glutamine, N-acetyl-l-glutamate, glutamate, aspartate, N-acetyl-l-aspartate, docosahexaenoic, icosapentaenoic, and dihomo-gamma-linolenate distinguished proliferative diabetic retinopathy patients from T2DM patients.^604^ Enrichment pathway analyses for major metabolite biomarkers indicated arginine biosynthesis metabolism, d-glutamine and d-glutamate metabolism were dysregulated in advanced stages of diabetic retinopathy.

Metabolic snapshot of COVID-19 revealed some additional interconnection pathways implicated in disease pathogenesis, including citrulline, phenylalanine and histidine, 2-aminobutyric acid, asymmetric dimethylarginine.^605^ The disordered metabolic pathways of primary Sjögren’s syndrome patients are associated with tyrosine metabolism, tryptophan metabolism, aspartate and asparagine metabolism, carbon fixation and affect neurological cognitive impairment, inflammatory injury, and the immune response.^606^ Pathway analysis by urinary metabolomic study demonstrated that aberrant metabolisms involved in aspartate metabolism, glycine metabolism, glycolysis, glyoxylate metabolism, and TCA cycle.^607^ Metabolic signatures enriched metabolic pathways of multiple myeloma patients were linked to amino acid metabolism and biosynthesis, and insight into elucidating disease pathogenesis.^608^ Characteristic biomarkers succinic acid semialdehyde, uracil, uridine or metabolic pathways enriched in lipid metabolism, amino acid metabolism, nucleotide metabolism and glycometabolism were identified and related to specific multiple trauma complicated with sepsis.^609^ Through untargeted analysis, a total of 120 candidate differential metabolites were detected in patients with ischemic stroke and markedly altered metabolic pathways were purine metabolism, steroid hormone biosynthesis, or CoA biosynthesis.^610^ Metabolic profiling using high-resolution mass spectrometry of cystic renal disease patients was collected and impact several pathways involved in purine and pyrimidine, aminoacyl-tRNA biosynthesis, glutathione, TCA cycle, etc.^611^

## Enabling precision treatment

There is not any specific therapy for satisfying all the patients. Thus, to predict the therapeutic response with matching the right patients at the right treatment is necessary in clinic. Additional techniques are critical to discover effective and potential biomarkers to guide patient management matching the proper treatment. Metabolite profiling as cost-effective and productive way enables holistic and systematic analyses of metabolites and can be utilized to predict and monitor the response to drug treatment, uncover therapeutic target for drug discovery, personalized management to reduce disease burden. Application of small metabolites to predict specific response to drug therapy is closely related to patient’s pharmacological phenotype and could generate more information than other omics data for interpretation of the metabolome data.^612–614^ Furthermore, it enables exploring promising models to predict therapeutic response.^171,615,616^ Small molecule metabolites can be used for diagnosis and prognosis of patients, predicting pharmacological responses to the peculiar treatment. Furthermore, metabolic signatures can provide the huge information from targeted metabolic pathways or precision drug therapy. Distinctive metabolite signatures that are useful for identifying different therapies responses are summarized (Table 1).

Irajizad et al. conducted plasma metabolomics profiles and artificial intelligence using a deep learning model to identify biomarkers for predicting response to neoadjuvant chemotherapy in triple-negative breast cancer.^617^ According to metabolic profiles, taurine, glutamine, glycine and hypoxanthine were potential biomarkers of ladder cancer patients treated with neoadjuvant chemotherapy and pathway enrichment analysis characterized significant alterations were related to amino acid metabolism.^618^ Amino acid metabolism seems to be a predominant pathway altered in ladder cancer patients and has potential value in enhancing the efficacy of chemotherapy. Notably, hypotaurine and taurine metabolism, pentose and glucuronate interconversions were the most altered pathway for subcutaneous immunotherapy.^619^ In this study, the authors found taurine, l-alanine, and hypotaurine, considered to be predictive biomarkers relevant with effective subcutaneous immunotherapy.

A recent study investigating the relationship between anti-VEGF therapy and serum metabolome and described differential metabolite LPC 18:0 may be a potential biomarker for guiding treatment options for macular degeneration and choroidal vasculopathy.^620^ It has been reported that decreased kynurenic acid in cerebrospinal fluid and kynurenic acid/kynurenine ratio represent a biomarker of epileptic spasms and further therapeutics method should be explored to increase the kynurenic acid level.^621^ Previous report has shown that after 4 weeks of olanzapine monotherapy in schizophrenic patients, methyl n-methylaminobenzoate as response biomarkers in the kynurenine pathway is associated with treatment outcomes.^622^ Metabolic profile alteration to molecular phenotype of psoriasis vulgaris patients showed that SM (d16:1/16:1) and Cer (d18:1/18:0) correlated with the biochemical indicators and could contribute to precision treatment.^623^ Another study used metabolomic profiling and small metabolites (N-methylisoleucine, nornicotine, 2,3-dihydroxybutanoic acid) were able to discriminate rheumatoid arthritis patients with early response to methotrexate therapy.^624^ These clinical applications of small metabolites provide excellent examples to illustrate new channels for targeted therapies and enabling precision treatment.

## Modulating metabolism

Modulating metabolisms with small molecules have been known for decades. Metabolic therapies are imperative and bring new opportunities for patients. Metabolic disorders are caused by various mechanisms. Recently, metabolism has acquired interest regarding the relationship with environmental factors, host genes and diseases.^625–630^ How does small molecule metabolites drive phenotype modulation? The common regulating mechanism of active pathway is metabolites bind allosteric sites on enzymes. Discovery of the relationship network or pathway of metabolite interaction can uncover the action modes of regulation. Numerous works have revealed differences changes in small molecular metabolites associated metabolic pathways are closely related to therapy efficacy and potential drug targets.^631–633^ Perhaps the application of these metabolic pathways involves small metabolites could better clarify the development of complex diseases in the future.

To exploiting the unique features of modulating metabolism with small molecules for treatment and monitoring is a very promising direction. The endogenous metabolite profiling provides the best view of disease phenotypes. Advanced screening approaches by analyzing the metabolic profiles have become increasingly application in metabolism study.^634–637^ Simultaneously, continuous development in high-throughput metabolomics technology has allowed considerable progress to be made in determining disease pathogenesis, understanding the various relationship between metabolic regulation and disease. Single-cell metabolomics technologies will reveal new insights into modulating metabolism with small molecules.^198,214,413,638,639^ It can provide meaningful cell phenotype, enabling us to analyze cell status and obtain the overall biological information.

Metabolic perturbations in vivo contribute to early discovery and mechanisms of phenotype modulation. Decoding the molecular mechanism of metabolic alterations will provide a promising way for novel therapeutic interventions. Metabolic alterations can modulate the cell signaling pathways to maintain the systemic homeostasis. The most diseases (e.g., obesity, diabetes, hypertension, or depression) have strong metabolic disorders, many chronic diseases (e.g., Alzheimer disease, or cancer) have unexpected metabolic basis of associations.^640–642^ It still needs a significant treatment window for effectively optimize therapies by precisely inhibiting the metabolic targets. To blocking metabolic pathways or inhibiting metabolic enzymes are almost impossible to generate an effective treatment. Metabolic disorders have become a feature of several cancers. Interestingly, several studies showed that targeting metabolic enzymes could significantly inhibit tumors to promote an effective therapeutic intervention.^370,643–646^

Major findings of previous studies in small molecule metabolites drive metabolism were summarized in Table 1. Targeted metabolomics identified energy metabolites of lung adenocarcinoma cells and found that KCNK3 can inhibit proliferation and glucose metabolism through activation AMPK/TXNIP pathways, indicating KCNK3 may be a potential therapeutic target.^647^ The authors examined a total of 202 relationship features between various cancers and metabolites, and showed gamma-glutamylisoleucine, 7-alpha-hydroxy-3-oxo-4-cholestenoate, gamma-glutamylleucine, and 1-oleoylglycerophosphocholine were the most dangerous metabolites for ovarian cancer, lung cancer, glioma and breast cancer, respectively. Analyses in these causal links demonstrated these small metabolites play a key role in phenotypic regulation to distinguish cancer patients in clinic.^648^ Pathway enrichment analyses indicated that the imbalance of purine and amino acid metabolism could affect the prognosis of patients with oral squamous cell carcinoma.^649^

A recent study conducting an inquiry into the relationship of small molecule metabolite hydroxyasparagine in blood samples associated with the progression of chronic kidney disease patients.^650^ Another study used serum metabolomic analysis and differentially expressed metabolites, such as triethanolamine, chavicol and alpha-methylstyrene, that involved in platelet degranulation and immune responses, and metabolism process were firstly identified as biomarkers in COVID-19 progression.^651^ A study suggested that the mechanism of lipid metabolism plays a critical role in pathological process of osteoarticular tuberculosis.^652^ Multivariate statistical analysis based on open database, metabolic differences of altered small metabolites were identified in superior limbic keratoconjunctivitis patients, and fundamental processes mainly involved in the inoleic acid metabolism, butyrate metabolism, ketone body metabolism, carnitine synthesis, and etc.^653^ Glutamate metabolism and urea cycle are related to psychiatric symptoms and accounted for the highest proportion in the altered metabolic pathway, and decreased in the schizophrenia group.^654^ A study has demonstrated that glycerophospholipid metabolism and arginine and proline metabolism pathways are related to inflammatory states and β-pseudouridine, may participate in inflammation regulation.^655^

## Functional target

Metabolite has a wide range of biochemical function, a growing area of researches is the usage of small molecule metabolites to discover the metabolic targets with optimal therapeutic response for precision medicine. A change of metabolite levels as results of the modified enzyme activities indicates a phenotype alteration because of metabolite concentrations provide a close association with biochemical activity. Endogenous metabolites as therapeutic molecules targeting regulators prone to modulate metabolism activity with key metabolic pathways such as regulating multiple enzymatic reactions. Recent advances in high-throughput metabolic flux analysis technologies using stable isotope tracer methods make characterization of a large scale of endogenous metabolite for characterizing and tracking the metabolic activities.^656–660^ It could provide potential therapeutic targets depending on the improved and detailed understanding of the interaction between metabolism in vivo and functional status. A clear understanding of molecular mechanisms about targeting central metabolic pathway always plays a key role in the discovery of drug targets for optimal therapies.^661–663^ Metabolomics directly contributed to uncover novel targets can elucidate disease mechanisms in various diseases. From a point of view of metabolism, such knowledge will uncover new therapeutic targets related to phenotypic variation. Understanding the metabolic dysregulation can facilitate drug development and provide therapeutic targets for disease therapy. Numerous active compounds as modulators of metabolism and could target metabolic regulation mechanism.^664–669^

The disordered metabolic pathways associated with COVID-19 patients performed by quasi-targeted metabolomics with pathway enrichment, and showed glutamine/glutamate ratio markedly related with severe disease.^670^ It is therefore proposed that elevated glutamate level is associated with the increased risk of disease infection. Nevertheless, elevated glutamine was associated with a reduced risk of severe. This study has provided the probable targets for COVID-19 patients. In another study, concerning plasma metabolites, Ozaki et al. found that small metabolites can predict the progression of cognitive impairment in Alzheimer’s disease.^671^ Metabolic profiling of cerebrospinal fluid revealed pentose phosphate pathway is an important target for sedatives to change brain metabolism.^672^ The study carried out by Thomas et al. highlighted pathophysiological mechanisms from serum metabolome, and demonstrated metabolic disruption of choline phospholipids as among the strongest predictors were associated with severity of traumatic brain injury patients.^673^ Furthermore, a study proven palmitic acid is a key metabolite as promising therapeutic target, which accelerates cellular senescence by producing living oxygen in kawasaki disease.^674^

According to Scarale et al., tryptophan, kynurenine and hexanoylcarnitine are associated to improve the mortality prediction of type 2 diabetes.^675^ Metabolomics and mass spectrometry analysis have identified succinate as a therapeutic target for aortic aneurysm and dissection.^164^ Metabolic alterations of colorectal cancer patients were assayed by functional metabolome profiling and glycodeoxycholic acid positively showed high specificity and sensitivity correlated with CRC.^676^ Further findings showed GDCA can promote cell proliferation and migration, and PARP-1 was identified as a key target. A study profiled metabolome and identified subtype-specific N-acetyl-aspartyl-glutamate as a key tumor-promoting metabolite and therapeutic targets for advance precision treatment for triple-negative breast cancer.^22^

Targeting tryptophan metabolism in cancer has curative potential. For instance, tryptophan metabolism is a major metabolic pathway which restricts anti-tumor immunity and promotes intrinsic malignant properties of tumor cells, and considers as a target for cancer immunotherapy.^677–679^ Tryptophan metabolism changes could result in a series of alterations in tumor microenvironment and tumor cells, and then promote tumor progression. Via hindering DNA repair, small molecule metabolites had accumulated in tumors involved in abnormal metabolism.^680^ Mechanistically, targeting isocitrate dehydrogenase 1 and 2 (IDH1 and IDH2) enzymes result in elevated levels of 2-hydroxyglutarate in cells and its accumulation boosts rapid development of tumors. Functionally, this better define relationships between small molecule metabolite disrupting DNA repair and biochemical function that benefit efficient treatment. In summary, these metabolic targets can enhance precise treatment in the upcoming era of precision medicine.

## Metabolic networks exploration

Metabolic perturbations could be modified by drug or natural products treatment as a crucial mechanism for its effects. Most natural products influence on multiple rather than single targets to exert the bioactivity. Both targeted and untargeted small molecule metabolites-based metabolomics have been used to characterize unexpected metabolic changes in biological samples, understand the metabolic processes and explore network targets and mechanism in various organisms.^681–683^ This analytical process always generates increasingly complex datasets at a large scale (thousands of metabolites), cause processing, analyzing, and interpreting relationships of small molecule metabolites are major challenges. Biological interpretation of the connected informative relationships of small molecule metabolites could be formalized as metabolic networks based on the prior knowledge, where the feature metabolites as nodes and the related metabolites are connected as edges.

Integrating multi-layer networks to use prior metabolic knowledge would help to improve the identification of metabolites and derive new interpretation of biological contexts.^684–686^ Once the metabolic networks of co-regulated metabolites are established, and then metabolism information will be mined using advanced algorithms. Importantly, the recent use of multiple network constructions and graph-based methods to perform topological analysis focused on analyzing the metabolic processes or metabolites data associated with a phenotype of interest.^687–689^ Metabolic networks or graph are generated depend on the prior biological knowledge. Network metabolites are co-regulated or connected within metabolic pathways. Instead, it represents the interconnections of metabolism network connected metabolites via distinct pathways. If correlation value of metabolites in a metabolic network are reaches a given threshold. Based on similarity or correlation of the identified metabolites, the graph analysis (e.g., metabolite graph and compound reaction graph), advanced statistical methods, and data analysis can be used to explore the inter-connected data to reveal metabolite relationship in biological samples.

During the past few years, network-based approaches towards multi-targeted compounds represent an important tool owing to its potential for ascertaining and investigating new drug targets and complex relationships.^690–693^ The ‘network pharmacology’ created by Hopkins and focus on a therapeutic concept from ‘one target-one drug’ to ‘target-network-component’ to combat the complex diseases.^694–697^ It used bioinformatics and high-throughput screening method to facilitate the prediction of various drug targets network based on the establishment of biological models, and is becoming more important in revealing the underlying mechanisms of drug actions. By analyzing the highly connected nodes in metabolic networks may open new avenues for discovery of mechanistically relevant signals for specific multi-target natural compounds. Thus, systems analysis of diverse metabolic pathways to identify novel targets may overcome pitfalls and facilitate change concepts of current drug design and develop new diagnostic as well as targeted therapeutic tool via exhibiting multiple targets and action modes.

Multi-omics interaction networks were constructed and showed that multiple biomarkers included pyridoxamine phosphate, folic acid, pyridoxal phosphate, and vitamin metabolism disorder was pathological characteristics of pulmonary tuberculosis patients.^698^ Based on 127 metabolic signatures from the Alzheimer’s Disease, specific metabolic networks modeling for diagnosis were constructed and provided key insights for personalized late-onset.^699^ Recently, Guo et al. performed a metabolic network-based identification modeling for mapping the differential metabolites, a panel of eight candidate metabolites (i.e., palmitic acid, pyruvate, tryptophan) were further indicated a high discrimination for non-small cell lung cancer (accuracy > 97.7%).^700^

## Efficacy evaluation

Metabolic profile change of complex diseases suggests distinctive aberrations of the metabolism can be due to drug’s treatment efficacy on patients’ genotype. Small molecule metabolite-based metabolomics plays an important role in discovering biomarkers to evaluate the efficacy of therapies and have become critical tools for investigating modes of drug action, identifying novel drug targets^701–704^ Particularly, by generating metabolic signature, it is increasingly being implemented to diagnose disease, monitor treatment and uncover the underlying mechanisms of complex diseases, seek to understand drug efficacy. Moreover, in-depth research on small molecule metabolite may guide drug efficacy, development, and safety. Selecting the most effective treatment drugs is an extremely important event. Identifying small molecule metabolites as biomarkers associated metabolic alteration by drug response before administration could greatly reduce costs of treatment. This is compatible with the notion that we need screening strategies of small molecule metabolite to determine each stage of treatment efficacy, and to develop more effective therapies. Clinical models combining small molecule metabolites have shed light on the search for biomarkers and therapeutic targets, could improve the accuracy of identifying patients.

The decrease of plasma kynurenine level may indicate the therapeutic response of escitalopram, suggesting that it may participate in the pathophysiological response of severe depression caused by escitalopram treatment.^705^ Metabolic signatures of cholangiocarcinoma patients showed that the TCA cycle was reversed, which was obviously manifested by the increase in the level of amino acid and citric acid as intermediate products of TCA cycle and have the ability to predict patients' response to chemotherapy.^706^ Stratification of methotrexate efficacy identified significant alterations to various metabolites such as phosphatidylcholines, glucosylceramides, sphingomyelins, hypoxanthine, etc, involved in nucleotide, energy, fatty acid/lipid metabolism.^707^ Serum-based metabolites involving L-arginine and arachidonic acid can serve as diagnostic biomarkers for breast cancer predicting therapeutic effects of trastuzumab.^708^

Medicinal plants usually depend on complex components are a great resource for treatment of metabolic disorders. However, due to complex components and multiple molecular targets, molecular action mechanisms of herbs and formulations are still not very largely clear. Usual methods are not enough sensitive to evaluate drug efficacy, even small effects of drugs can be sensitively detected by small molecule metabolites as disease-related biomarkers in clinical trials, by monitoring differences of metabolite profiles. Knowledge about metabolic regulation mechanism by herbal medicines can help to predict and understand the efficacy and toxicity. Recent years some studies utilizing metabolomics to elucidate the biological basis and mechanism of the effect.^709–711^ It focuses on fluctuations of small molecule metabolites and insights into drug efficacy assessment and investigates molecular mechanism of herbal plants as adjuvant therapy for aberrant metabolism-related diseases. Research has shown that small molecule metabolite-based metabolomics can be further used to identify the active compounds and targets, which develop new therapies.^712–715^

## Active ingredients discovery

Over the past decades, more than half of new drugs and drug leads have been developed from natural products that possess immense chemical structure with various biological properties. Recently, natural products in medicinal plants, such as alkaloids, flavonoids, terpenoids, carotenoids, and glycosides, possess therapeutic effects and are used as new therapeutic drugs.^716–718^ In clinic, herbal products are combined with conventional drugs to improve pharmacological effects. Active ingredients or drug leads from natural products have been a key source but their identification is always a challenge due to their complexity. Understanding effective mechanism of natural products or their derivatives or synthetic mimic requires elucidation of pharmacological response to complex phytoconstituents. Although a great advance achieved, one major challenge in discovering new active ingredients is unclear pharmacological mechanisms. The action mechanisms and efficacy profiles of herbal medicines for their potential use should provide in-depth information on elucidating the underlying mechanisms for active ingredients discovery.

Medicinal plants such as herbal extracts, formulae, and different compounds showed the pharmacological effects through regulating metabolic disorder and mechanism pathways due to multi-compound interactions and diverse chemical structures. High-throughput metabolomics agrees with holistic view and insight into a comprehensive mechanistic efficacy of herbal medicines, including medicinal plants, preparation, active compounds, aqueous extracts and formulas or patent medicines.^719–723^ Target small molecule metabolite based-screens offer numerous advantages for functional ingredients discovery from natural products as a treasure trove for drug development, and allows in-depth understanding of the possible targets and action mechanisms. Advanced metabolomics techniques consist of LC-MS, NMR, and GC-MS in combination with pattern recognition analysis or multivariate statistical analyses could identify a large number of metabolites and impact on diagnosing disease, discovery of biomarkers, investigation of phenotypes, classify physiological status and response to treatment, unravel efficacy of metabolic-targeting drug, cover the full pipeline of lead compound discovery and development from medicinal plants.^719,724–727^ Based on multiple metabolic alterations involved in disease pathogenesis, it is particularly pivotal to explore herb-derived bioactive ingredients for these mechanistic basis, damaged metabolic pathways and therapeutic targets of metabolic diseases. Moreover, herb-derived bioactive ingredients have been screened and validated in vitro and in vivo, to investigate the underlying changes of small metabolites and metabolic pathways, and to find potential targets (Fig. 6). They can provide a functional relationship between chemical diversity and metabolite changes.

Natural compounds as potential therapeutic agents have gained increasing interest due to ability to target metabolism and their diverse structures. Different metabolites involved in metabolic alterations could be targeted by the active components due to their efficacy in the clinic. Various herb-derived bioactive compounds could target the metabolic regulation mechanism of diseases and exhibit therapeutic potential. Cell culture and animal model experiments had been to analyze the potential effect and metabolic activity, the additional clinical studies are necessary to fully elucidate therapeutic efficacy and mechanisms of action. High-resolution prediction technology or visualization approaches enhances screen and validates the lead compounds from natural products.^728^ Eighty-nine compounds were identified and calceolarioside B, isoacteoside, and 2'-acetylacteoside being validated to treat renal fibrosis. The functional mechanisms modulate the metabolic pathways or whole metabolism of natural bioactive compounds needs to be elucidated for use as therapeutic agents. Bioactive compounds could target the small molecule metabolites associated metabolic process of a specific phenotype and modulate metabolic activity of distinct pathways, hold great potential as therapeutic preparations for highly complex diseases.

The potentially vasodilative compounds from *Uncaria* were screened as isocorynoxeine, corynoxeine, rhynchophylline, isorhynchophylline, by correlation analysis of small metabolites.^729^ In vitro and in vivo constituents of American ginseng were in-depth investigated using mass spectrometry, and then natural bioactive compounds associated with therapeutic effects were explored using correlation analysis between in vivo constituents and marker metabolites, and revealed ginsenoside Rd, and pseudoginsenoside F11 may be potential active markers of American ginseng.^730^ By correlation analysis between anti-inflammatory activity of Scutellariae radix and small metabolites, a total of ten potential components were screened out with high correlation coefficients. An in vivo study revealed oroxylin A had the potential effect of antisepsis by inhibiting TLR4/NF-κB signaling pathway.^731^

## Metabolic process of active components

Since the beginning of the 19th century, natural products such as morphine isolated from opium plant have been explored in drug development. With the concept of returning to nature, natural products have attracted great attention and are useful agents for lead compound discovery and new effective drugs. The herb-derived phytochemicals known as natural products have health benefits and their activities and the underlying mechanisms have remained elusive, due to lack a method about how to characterize active components to the whole effect that visualizes dynamic changes in vivo and these components possess diverse effects. To reveal the pharmacological effects of herb-derived phytochemicals with multi-components and multi-targets, the following issues should be solved with emphasis: how to detect active components with low content, elucidate metabolic pathway, reveal overall in vivo metabolic process and effective mechanism. Considering complexity, meanwhile it has numerous metabolic reactions in vivo, producing diverse metabolites. Metabolic processes study in vivo is important to determine multi-component characteristics of efficacy and guide the new drug development, or speed up drug discovery from natural products.^732–734^

Drug discovery process from natural products exhibits some obstacles that presented by the extraction, purification and separating active components. Several of the obstacles have been addressed by employing small molecule metabolites-based screening. Metabolic processes undergo biotransformation mediated by phase I and II reactions or gut microbiota direct impact on the efficacy and are important for determination of pharmacokinetic parameters on the concentrations of active components to the organs and tissues over time.^735–737^ To reveal in vivo processes (absorption, distribution, metabolism, excretion) of multi-active components, it can elaborate the efficacy material basis. Metabolic parameters in vivo, C_max_, T_max_, t_1/2_, and AUC_0–t_ were the most calculated. Active substances are from the prototypes and the metabolites entering into human circulation, which is directly related to the metabolic process. Endogenous small molecule metabolites can be linked to specific metabolic phenotype, activities, or functions, are closely linked to therapeutic efficacy to screen out the key components in the whole in vivo process.

Understanding metabolic fate of active components is a key factor for elaboration of new therapeutic agents. However, the identified herb-derived bioactive metabolites suggesting the curative potential by modulating multiple targets of disease-associated networks. Owing to the high sensitivity and stability, modern mass spectrometry coupled with all kinds of hyphenated chromatography separation techniques has a pivotal role in the exploration of in vivo metabolism of active components.^738–757^ Cheminformatics utilizing computer-aided, high-throughput virtual screening, network-based and machine learning techniques have opened up a new avenue in exploiting naturally-inspired products for lead compound and active components discovery.^758–767^ A non-targeted metabolomics screening strategy is carried out focusing on the in vivo metabolites that exist in the administrated samples and do not exist in blank biological samples, by exploring the dosage-effect relationships. To selecting in vivo metabolites being associated with therapeutic effect as pharmacological index reflecting overall efficacy is of great significance. Simultaneous determination in vivo of herbal components is technically challenging due to complex interactions with co-existing components. Indeed, LC-MS with selective reaction monitoring mode or background deduction method and pattern recognition analyses were adopted to eliminate possible interference as an effective technique for the identification and quantitative analysis of in vivo components in biological media. Due to active components are complex and their contribution weight to effect is different, it is necessary to explore in vivo metabolic processes of combination multiple active components based on AUC-weighting approach, so that can guide practice administration in the clinic.

Metabolic whole-process in vivo could be realized by efficiently constructing the relationship among endogenous metabolites and compounds. Based on different scores of relationships building in vivo metabolic network, active components markers including metabolites or prototypes that are highly related to small molecule metabolites can be effectively screened out by molecular network technology.^768–772^ To screen potential active candidates for revealing overall effects, compounds with highly relevant and large VIP values (>1) rankings were selected and identified by the correlation analysis model. The appropriate mix of active components could be optimized via metabolic phenotypic screening and their targets and molecular mechanisms can be revealed by network pharmacology, artificial intelligence, or computer docking.

## Metabolic homeostasis and gut microbiota

Increasing evidence shows that homeostasis balance of the human body depends on reciprocal interaction with gut microbiome. Microbial dysbiosis is a contributing factor for onset and progression of diseases. Gut microbiota contribution to control homeostasis, modulating immunity environment, and maintaining systemic health is an emerging.^773–776^ It produces a large number of small metabolites that regulates host metabolism responses and metabolic disorders. To date, there is growing evidence that most of small molecular metabolites have beneficial impacts on the host.^777–782^ Gut microbiota can produce various metabolites as messengers between host and microorganisms. Major communication between host and gut microbiota takes place through the metabolites, such as acetate, butyrate, and propionate, and via the microbiota composition modulating metabolism processes.^783–786^ Gut microbiota acts as an “invisible organ” and produces active metabolites via their receptor signal to regulate the host metabolism that affects systemic health, and plays a variety of effects on the host from shaping gut structure and function to the modulation of the host status. The increasing evidence showing that gut environment disorder could affect various organs lead to metabolic diseases.^787–790^ Imbalance of gut microbiota is closely associated with disease mechanisms, and implying a new therapeutic avenue.

Probiotics can improve the intestinal microecological balance of the host, and play a positive role in enhancing the immunity of the body and helping the absorption of nutrients. The host also could produce a variety of important metabolites that also affect the balance of microbiota. Gut microbiota by producing various bioactive compounds is linked to pharmacological effects and plays an important role in drug absorption, metabolism and efficacy.^791–794^ The gut microbiota as an indispensable “organ” to regulation of drug metabolism affects the inherent bioavailability of drugs to reduce toxicity and increase the target efficacy. It can activate or inactivate the pharmacological effect of natural products. Natural products can alter the microbiota compositions or their metabolites via modulating the host metabolism, which could enhance therapeutic effects and attenuate adverse reaction in pharmaceutical development. For instance, some metabolites as ligand metabolic signaling can activate cell-surface GPCRs and may provide potential targets.^795^ Herbal ingredients could regulate the metabolic disruptions by altering gut microbiota, particularly to reveal the dysregulated metabolites in metabolic pathways interacting with gut microbiota. Since natural products are metabolized and mainly absorbed in the intestinal tract, the secondary metabolites could regulate metabolic perturbations. Microbial-derived products receive considerable attention in disease treatment based on their efficacy by modulating expression of metabolic regulators.

**Fig. 7:**
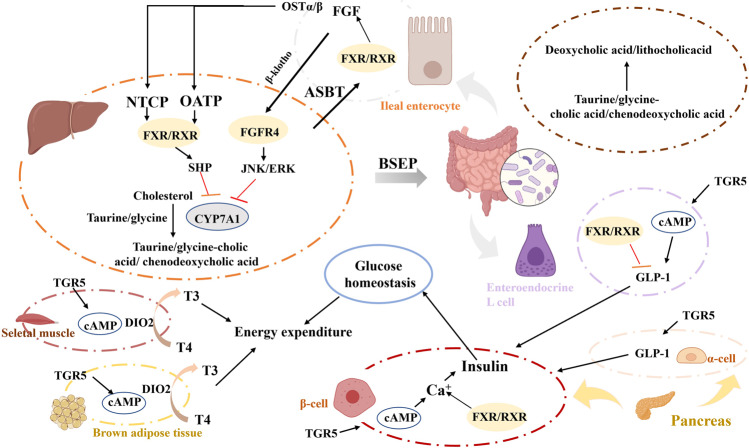
Schematic summary of interactions of bile acids and gut microbe participate in the host metabolism. Note: BAs, bile acids; BSEP, bile salt export protein; FGF, fibroblast growth factor; FGFR, FGF receptor; RXR, retinoid X receptor; NTCP, sodium taurocholate cotransporting polypeptide; OATP, organic anion-transporting polypeptide; SHP, small heterodimer partner; JNK, c-Jun N-terminal kinase; ERK, extracellular signal-regulated kinase; T3, thyroid hormone; T4, thyroxine; DIO2, type 2 iodothyronine deiodinase; ASBT, apical sodium-dependent bile acid transporter; OST, organic solute transporter. Primary bile acids are synthesized and then conjugated with taurine or glycine in hepatocytes. Conjugated bile acids are transported into the bile duct by BSEP. Most conjugated bile acids are reabsorbed via ASBT and circulate to the liver by OATP, OSTa/b, and NTCP. Bile acids acts as the endogenous ligands for FXR and TGR5 to generate distinct effects on metabolism regulation. The figures created by BioRender

Human metabolism is also influenced by regulating gut microbiota that could regulate metabolic products of the host metabolism enter into circulation system of host and participate in metabolic regulation mechanism in vivo. It continues the awareness of gut microbiota influences on metabolism, due to the complexity and interplay between the host gut and microbiota. Trimethylamine as a toxic molecule cross the gut-blood barrier for circulatory system homeostasis of cardiovascular diseases.^796^ Gut microbiota as signal distant organs via the systemic circulation affects the pathological processes on controlling host vascular and energy homeostasis. Due to gut microbial dysbiosis, disorder of energy homeostasis balance has a key role in the disease progression. Some evidence points to the important role of gut-derived metabolites in regulating energy metabolism.^797–802^ For instance, SCFA as ligands activating cellular signaling cascades have a variety of beneficial impacts in regulating energy metabolism, especially in obesity-related diseases.^803^ Small metabolites of gut microbiota, such as bile acids (BAs), and amino acids could decrease the insulin sensitivity and regulate metabolic dysfunction and immune homeostasis, which play a crucial role of the glucose regulation. Interactivity relationship of gut microbiota and BAs as signaling regulators have a profound impact on disease progression through modulating metabolic homeostasis (Fig. 7). In addition, many works have shown that relationship between gut microbiota and BAs plays a critical role in the systemic homeostasis.

Reciprocal relationship between host and gut microbiota to maintain homeostasis of biological processes is in dynamic balance and that influenced by endogenous metabolites or biological effects of their precursor. Gut microbiota could produce various small molecules and metabolites, plays a physiological role in maintaining hot homeostasis and internal stability through its metabolites serve as messenger affecting disease states. In addition, some metabolites are only released from gut microbiota, such as bacteriocins, short-chain fatty acids, etc. It showed that tryptophan could also be metabolized by gut microbiota, leading to synthesis of bioactive indoles.^804^ Additionally, gut microbiota metabolizes primary bile acids, showing a protective role in gastrointestinal diseases. Gut microbiota and their metabolites, as a whole, have been considered as versatile “organ” maintaining the body homeostasis. As gut microbial metabolites, such as secondary bile acids and short-chain fatty acids, could alter the metabolic fluxes by interacting with host receptors, thereby significantly affect metabolic homeostasis leading diseases.^805–807^ These metabolites participate in diverse metabolic processes, such as cell communication, energy metabolism, and host immunity, could influence human physiology, and future studies should define the functional relevance involved in disease pathogenesis.

Gut microbiota plays an important role in affecting the metabolic homeostasis. It has been increasingly appreciated small molecule metabolites-based metabolomics integrating with gut microbiota to explore systemic interaction between metabolites, gut microbiome and disease subtype. Small metabolic biomarkers have used to analyze the interaction between gut microbial composition and metabolism via omics analysis. Recently, Brial et al. discovered that hippurate was a key co-metabolite of host-microbial, could mediate the metabolic improvements associated with high-richness microbiota.^808^ This work has provided a beneficial biomarker hippurate as a mediator of metabolic health that contributes to metabolic improvements in terms of metabolic phenotype control for the host. In a recent study in Nature, Wu et al. had discovered host-microbe interaction that microbiota-derived inositol phosphate metabolism contributed intestinal homeostasis through mediating the HDAC3 activity in the intestine.^809^

Analyses in airway serum composition and microbiome datasets demonstrated the gut microbiota can influence on metabolic activity.^810^ Through depicting the overall landscape of metabolome and microbiome in rheumatoid arthritis patients, 26 genera and 41 metabolites were remarkably altered and function prediction model observed the depleted dysregulation pathways of amino acids biosynthesis.^811^ Integrated metagenomic and metabolomic analysis had characterized the interactions between metabolites and gut microbiome in early-onset colorectal cancer patients and helps explain the disease pathogenesis. Microbiome-derived metabolites such as bile acid, tryptophan, and choline could be used for the accurate and rapid detection of disease.^812^ Changes in the levels of several amino acid derived metabolites in a cohort of obese patients, such as the decrease of phenylacetylglutamine and the increase of L-histidine, linked to changes in the gut microbiota composition and function.^813^ Particularly, *P. pentosaceus* and *L. lactis* can ameliorate NAFLD progression by regulating tryptophan metabolism of the gut-liver axis and also closed associated with metabolic dysregulations of bile acid and indole.^814^ A study analyzed the correlation between serum metabolites and gut microbiota in elderly patients with chronic heart failure insights into metabolic phenotypes.^815^ Further, biocytin was negatively correlated with *Escherichia Shigella*; lactose and sucrose were negatively correlated with *Haemophilus*; bilirubin was positively correlated with *Klebsiella*; inosine and riboflavin were negatively correlated with *Klebsiella*. From the plasma metabolome analysis, a positive correlation between metabolite levels and the amount was observed and provide a guide for modulating gut microbiome may help shape a healthy metabolome.^816^

## Influence factors

Duo to the influence of metabolism is multifaceted factors including exogenous or endogenous. Small metabolites could be widely varied by sex, age, weight, nutrition, medications, lifestyle, and circadian rhythm. If they changed, accordingly, the metabolism will also change and then different small metabolites will produce. Certainly, these factors are impossible or even difficult to monitor and may represent the most challenging in metabolic biomarkers studies. These factors can lead to metabolic dysregulation of body, which can cause metabolic pathway alterations in disease-associated patients. When some influencing factors were intervened by drugs, early prevention, timely and effective disease treatment can achieve.

Small metabolites could participate in the entire “metabolic chain” in body, and has important implications for identifying the metabolic features related to disease phenotypic variation. Although the potential metabolite markers for diagnosis and treatment of diseases have screened and made great progress, and still needed to be validated the feasibility in a large number of cohort studies. Differences in some marker metabolites between the humans and animal experiment need to consider it. Compared with animal experiments, some elements, e.g. gender, age, psychological status, disease history, diet, exercise pattern, habits and customs, and other lifestyle differences have been considered to be significant variation factors for the altered metabolism within the body.

Metabolome is affected by genetic factors, intestinal flora and environmental exposures.^817–820^ These changes might originate from the environment, physiological or pathological status, diseases, drugs or other external stimuli, which can significantly contribute to the metabolome composition. Moreover, small molecule metabolites-based metabolomics can identify as many marker metabolites as possible and then reveal the metabolic network associations of bioactive substances and effective targets, needs to validate these associations, and further mechanistic research on dysregulated metabolites should be implemented and reproduced. Metabolomics contrary to genomics, proteomics, or transcriptomics, can rapidly and accurately reflect a more clearly phenotypic state of organisms, however it influenced by outside confounding factors. Previous studies have shown these factors could influence the metabolic phenotypes of metabolism-related diseases.

## Limitations and challenges

As we have shown, small molecule metabolites-based metabolomics has enormous potentials and many applications, however, several challenges and limitations need to be further addressed. The complexity of a large number of metabolic signatures that present in “dark metabolome” associated with manifestations of transient phenotypic state is one of the huge challenges. Advantage of omics as tools for biomarker discovery simultaneously quantifying a large number of small molecules in biological media. Since metabolome associated metabolic alteration is highly complex, and no uniform strategy and distinct analytical platform can analyze all the small metabolites of the whole phenotype. Others are the challenges in small metabolite analysis associated metabolic alteration include the genetic factors, environmental factors, or gut microbiota. Numerous small metabolites from different biological media were considered to be candidate biomarker of predicting the of disease onset, and therapeutic response. Determination of small metabolites as excellent candidate are measured from no-invasive samples such as urine and blood. Potential role of the target metabolites needs further verify and identify as biomarkers in the disease diagnosis, management and prognosis of diseases. Predictive ability, disease prognosis and diagnostic value of the established metabolic profiles need further validate with larger sample sizes in real-world medicine. In addition, method standardization of large randomized clinical trials is needed.

Previous studies have shown that quality of metabolic data lead to significant variability that influenced by sampling techniques and analytic approaches.^821–823^ Technological limitations or insufficient use of metabolomics are partly caused by structurally diverse metabolites, standardization and uniformity of instrumentation, temperature variation, sample preparation and handling, proficiency and availability of trained staff. It is important to emphasize the standardization of laboratory procedures, such as extraction, sample processing, and other analytical protocols, is a fundamental step to obtain biologically meaningful metabolic results. It is necessary to establish the commonly accepted standardized criteria or protocols for sample extraction, data mining and data reporting. This could resolve the major challenges with metabolite identification by used various spectroscopy, chromatographic methodology to influence specific constituent, result evaluation by employed different statistical methods and interpretation of clinical significance, all of which could affect experimental or clinical outcome and thus limit the application of small molecule metabolites-based metabolomics into clinical aspects. In the past decade, the significant progress and improvements in technical aspects have been made for small metabolite analysis from metabolic perturbations in tissues and biofluids to further promote understanding of molecular mechanisms to advance meaningful interpretation of metabolic features related to phenotypic variation. Analyzing and revealing metabolic changes in disease response to drugs could provide opportunities to discover the potential targets and biologically meaningful metabolic pathways for metabolism-related diseases therapy. Fortunately, targeted metabolic profiling of some metabolites has been endorsed to be applied in clinical practice for disease markers and potential targets identification for monitoring, diagnosis, and drug efficacy.

The accurate masses, fragment mass spectra and retention time should be provided to identify metabolites via database-based search methods. However, considering that significant amounts of datasets, special statistical software, complexity of computational processing, bioinformatics tool, lead to detecting specific molecules, validate the pathways and associations, analyze data even more difficult. Databases for metabolome analysis with extensive metabolite coverage with help of multivariate analysis have been significantly developed for data identification and visualization. Small metabolites are downstream of transcriptome-proteome and their metabolism were affected by various microbiota in vivo, so that multi-omics can create approach to explore the interactions of proteins, metabolites, genes, and microbiota, and then reveal the pathophysiological mechanisms in both diseased and non-diseased states. Fortunately, integration with other omics could insight into the characteristic metabolic alterations.^824,825^ Integrative analysis of omics data by multi-omics technology could provide the mechanistic insights into diseases and bring precision treatment.

One of the biggest challenges is mainly in the realm of data integration still in early stages and needs additional consideration. To achieve this goal, high-throughput integration multi-omics with help improvement of computational and bioinformatics techniques has greatly contributed to accurately identify the relevant small-metabolites and their biological processes involved in metabolic perturbations in vivo. Such integration analysis approach can effectively use it to understand abnormal biological mechanisms in the underlying metabolic network of interest by visualizing metabolic pathways. The integration importance of metabolomics with other omics is seen in some very recent research.^826–828^ The high complexity of metabolome poses another challenge for identifying metabolic features related to phenotypic variation. Fortunately, computational approaches and artificial intelligence-based algorithms representing promising tools for biomarker discovery is provided to overcome the above problems. Advanced analytical techniques, such as artificial intelligence, computational algorithms, metabolite imaging, statistical, and big data mining are urgently needed to improve coverage of the low-abundance metabolites for clinical validity and utility of small molecule metabolites involved in disease pathogenesis.

The full validation stage of the small molecule metabolites-based metabolomics workflow is missing and prohibits the biomarker discovery to clinical translation over time. However, there are several limitations should be addressed in future research. Due to lack of standardization in process research and external validation and some works need to be done before ascertaining biomarkers for clinical practice. Utilization of standardization process should be embodied in each stage, such as patient enrollment, sample collection and processing, storage, preparation treatment, data acquisition and in-depth analysis. In addition, the standardization of sample preparation and processing, data analysis and variation factors will help to promote the research of small molecule metabolites to beyond the discovery stage of biomarkers and towards the development and validation of clinical trials.

Indeed, small molecule signatures provide crucial information for diagnostic and prognostic biomarkers, and therapeutic response. However, it remains a subsequent challenge of translation of small molecule signatures from laboratory results to clinical and industrial application. An open issue is most researches still have done with smaller cohorts, and it requires future studies with a larger cohort dataset from multicenter studies in clinic should include many metabolite biomarkers and metabolic pathways for accurate diagnosis of diseases and better understanding metabolic alterations, and should be addressed in the future.

## Future outlook

Altered metabolism leads to characteristic metabolic phenotypes as the hallmark of disease that drive identification of new targets related to metabolic regulation mechanism could be applied for developing effective screening strategies for predicting early disease, or evaluation treatment monitoring responses. Metabolomics, as mentioned above, is a relatively young discipline relative to other omics, has identified small molecule metabolite biomarkers for the disease diagnosis, prediction, screening, and monitoring treatment. Compared to genome or transcriptome, coverage of metabolome remains limited and lead inadequate interpretation of the final results. Due to no single technology can offer an entire metabolic spectrum, thereby different advanced analytical chemistry platforms are recommended to integrate metabolomics with upstream omics and network target analysis. Integration of multi-omics datasets can represent a powerful method to reveal metabolic signatures related to phenotypic variation of patients.^829^ Moreover, multi-omics integrative analysis can uncover disease biomarkers and new pathological pathways, deepen understanding of mechanistic basis and therapeutic targets of metabolic diseases, and accelerate new drug development for better therapy, significantly enhanced translational capability.^830–833^ The integration of multi-omics analysis may present precise metabolic biomarkers, a global metabolic snapshot and metabolic networks, which can deepen exploration of underlying mechanisms towards improving clinical management of disease. However, due to the complexity of metabolic pathway data and the interaction between metabolic network and other factors, the integration of multi omics data is a huge challenge. It needs to establish an international network or a different platform with modern instruments for the integrative multi-omics data by biologists, statistician and chemists.

Future work should strive to solve many of the following clinical problems, e.g. limited in sample size and control groups, validation of candidate biomarkers in clinical settings. It is still difficult to rapidly separate the small molecule metabolite while keeping their metabolic states, especially under metabolic disturbance, because metabolite biomarker is susceptible to change of environmental factors. Future studies should validate the selected small metabolites in larger sample datasets to increase the analysis credibility and statistical power that help to our understanding of the aberrant metabolism. In this scenario, it needs to improve the detection precision and accuracy of small metabolites from the entire metabolome in real time analysis. Particularly, break the technical bottlenecks by substantial efforts needed to acquire high sensitivity and accuracy for extensive coverage detection of metabolome. Recently, high-resolution mass spectrometry improves detection capability and enable us to identify metabolic biomarkers towards clinical validation.^834–836^

Forthcoming research of diseases will address questions about the integrated metabolic phenotypes, and then how to transform them into clinical applications for better therapies. Large, prospective clinical practice can validate the discovered small molecule metabolites with high translational chances for diagnostics, prevention and therapeutics. Nevertheless, upcoming challenges will include harmonization and normalization of disparate datasets, protocols standardizations and new algorithmic analysis to better explore the underlying mechanisms and insights. Likewise, software for data processing and interpretation is becoming standardized and widespread. Standardization of procedures for meaningful and accurate management of metabolite biomarker research that modulates biological processes should be further refined for untargeted or targeted analysis towards ensuring laboratory results become clinical translational. Therefore, targeted and functional analysis approach to overcome limitations of conventional metabolomics is a new strategy for exploring the small-molecule metabolism associated mechanisms of complex alterations of systemic homeostasis. A large number of small metabolites have been identified as disease biomarkers or predictors.^466,837–842^ Therefore, future research should reduce the number of diagnostic or prognostic biomarkers to the most appropriate number.

With help of the enhanced AI or big data analytics, machine learning or developing algorithms, a clear understanding of underlying pathological pathways and metabolism-related diseases can offer a tangible route or evidence to support clinical decisions in addressing the formalized utilization of small molecule metabolites or metabolic phenotyping into routine clinic. On localizing metabolic alterations is key to driving our understanding of disease, advances in metabolic imaging in an unprecedented approach actively realized better resolution of metabolic property to quantify metabolites and illuminating metabolic pathways. In the near future, the significant improvement in metabolic imaging tool combined with innovative algorithms enables to differentiate tissue states for various diseases. Current applications of multi-omics via modern imaging techniques and computational improvements translate to clinical diagnosis, prevention and precision treatment, and facilitate selective treatment in future medical care.

Future work should explore the non-invasive, specific biomarkers with high diagnostic value, simplify the evaluation process assessment of candidate biomarkers into clinical application, develop new algorithms or bioinformatics software for exploring molecular interactions associated metabolic alteration, and establish effective association integrating laboratory and clinical results. Additionally, the further characterization of small molecule metabolites associated gut microbiome could shed light on metabolic features related to phenotypic variation of disease pathogenesis, enable to assist diagnosis, prognosis, treatment strategy selection, and realize the benefits that of small molecule metabolites bring to precision treatment. Small molecule metabolites-based metabolomics has greatly changed biomedical research. In the future, metabolite biomarkers will need to be effectively validated and transferred to clinical applications, thereby researchers should work closely with clinicians. For increasing the accessibility of metabolite biomarkers, its analytical instruments should become far cheaper and simpler, insights into specific metabolic phenotypes. With the joint efforts of the government and industrial community, all this will be achieved.

## Conclusion

Abnormal metabolites could serve as potential biomarkers for evaluating diagnosis and monitoring treatment response and prognosis, will provide abundant evidence for future precise medicine. A variety of metabolic pathways are altered in human diseases, including fatty acid oxidation, amino acid metabolism, lipid and energy metabolism, glycolysis, phospholipid metabolism, and tricarboxylic acid cycle that maybe considered as potential targets for further clinical trials. Metabolome has provided a comprehensive, dynamic, and precise picture of metabolic phenotype that could confer the personalized clinical practice. Metabolomics has changed the world of metabolite biomarkers, for its utilization of identification of pre-disease states, diagnosis, subtyping, prognosticating and monitoring treatment response, provide evidence for the early diagnosis, prevention, and mechanistic exploration. Current milestone findings encourage further investigations to urgent application in the clinic. Numerous studies are also going on metabolomics-based discovery of identifying the unique metabolic signatures, opens up the complex metabolite networks in physiological or pathophysiological processes; its biomedical applications can already be foreseen to monitor health and disease, assess disease severity or drug development, predict the time-course, monitor progression of diseases, and predict potential treatments, and elucidation of disease mechanisms.

The present review summarized the outcomes of most significant researches to extend the knowledge of small-molecule metabolite biomarkers. A major limitation is the absence of validation of metabolic phenotype-related mechanistic targets of diseases, which leads to lack the focused therapy and become increasingly prevalent. It is vital that we should dig out the most sensitive and accurate, specific metabolite signatures and conduct more studies to corroborate and validate these findings. However, standardization studies of metabolite applications methods and validation with a greater degree of certainty in large-scale clinical samples are needed before these tests could be a wide range of applications in clinical settings. In addition, multicenter exploration with large-scale populations in validation of small-molecule metabolite biomarkers is still needed for clinical translation and utilization. Integration multi-omics data combined with clinical measures has potential to facilitate delineating of disease progress and treatment. The combined analysis of multi-omics data focused on the precise metabolic phenotype characterization offer opportunities to facilitate deciphering the molecular changes underlying metabolic mechanisms in human diseases. Upcoming research should improve the diagnostic ability of potential biomarkers to easily predict diagnosis and prognosis. Additionally, future direction to address clinical application relates to the establishment of the relationships between metabolic profiles and clinical parameters. Future endeavors should increase the confidence in metabolite identification by systematic large-scale profiling analysis and emphasize determining the applicability of metabolomic-derived biomarkers and their clinical utility in large-scale clinical settings. Further targeted metabolic profile is needed to better explore the suitability of small-molecule metabolite as initial indicators of diseases, better understanding of pathophysiologic mechanisms, mitigating the risk and benefit from the best treatment, may open novel avenues for future precise medicine.
